# *Tripterygium* glycosides tablet ameliorates chronic kidney disease via gut-derived uremic toxins reduction in rats: alkaloids as major toxic ingredients

**DOI:** 10.3389/fphar.2026.1739319

**Published:** 2026-07-03

**Authors:** He Gong, Sixian Chen, Zhuohua Huang, Hong Yuan, Jiang Ma, Xin He

**Affiliations:** 1 School of Chinese Materia Medica, Guangdong Pharmaceutical University, Guangzhou, China; 2 Key Specialty of Clinical Pharmacy, The First Affiliated Hospital of Guangdong Pharmaceutical University, Guangzhou, China; 3 Guangdong Provincial Key Laboratory for Research and Evaluation of Pharmaceutical Preparations, Guangdong Pharmaceutical University, Guangzhou, China

**Keywords:** alkaloid, chronic kidney disease, constituents knock-out/knock-in technology, gut-derived uremic toxins, *tripterygium* glycosides tablet

## Abstract

**Objective:**

*Tripterygium* glycosides tablet (TGT), the major preparation of *Tripterygium wilfordii* Hook. f (TWHF), is widely used in treating chronic kidney disease (CKD). However, its complex composition and overlap between active and toxic constituents often lead to adverse effects, limiting precise clinical use. This study aimed to clarify the therapeutic mechanisms of TGT in CKD and identify its potential toxic ingredients.

**Methods:**

Using constituent knock-out/knock-in technology and HPLC fingerprinting, *Tripterygium* glycosides tablet was divided into an alkaloid-enriched fraction and a non-alkaloid-enriched fraction An adenine-induced CKD rat model was used to evaluate efficacy and toxicity. Non-targeted metabolomics and 16S rRNA sequencing identified key metabolites and gut microbiota, while a UPLC-MS/MS method quantified gut-derived uremic toxins.

**Results:**

TGT reduced serum creatinine, urea nitrogen, and uric acid levels, improved renal filtration, and restored kidney morphology. Metabolomic and microbiota analyses revealed decreased indole and amino acid derivatives and regulation of *g_Bacteroides* and *g_Rodentibacter*. UPLC-MS/MS confirmed reductions in indoxyl sulfate (IS) and *p*-cresyl sulfate (pCS), mainly via inhibition of the tryptophan–indole–IS pathway. Conversely, AEF increased creatinine, urea nitrogen, cystatin C, and trimethylamine N-oxide (TMAO), exacerbating glomerular dysfunction.

**Conclusion:**

TGT exerts renoprotective effects partly by modulating the gut–liver–kidney axis and reducing gut-derived uremic toxins, while alkaloids likely contribute to its toxicity. These findings offer new insights into the safe and effective use of TGT in CKD therapy.

## 1 Introduction

Chronic kidney disease (CKD), as a progressive condition, is characterised by alterations in the structure and function of the kidney caused by a variety of reasons (Kalantar-Zadeh et al., 2021). Approximately 10% of adults worldwide are afflicted with some form of chronic kidney disease (CKD), which causes 1.2 million deaths annually (Xie et al., 2018; Bikbov et al., 2020). According to the Sixth China Chronic Disease and Risk Factor Surveillance, the prevalence of CKD among Chinese adults was 8.2%, and the awareness of CKD was only 10% (Wang L. et al., 2023). The global prevalence of CKD is rising annually, and it is projected that CKD will rank as the fifth biggest cause of mortality worldwide by 2040 (Foreman et al., 2018).

*Tripterygium wilfordii* Hook. f (TWHF) has an extensive history of clinical utilisation in China (Zhang et al., 2020). Many studies have shown that TWHF has anti-inflammatory, anti-oxidant, anti-tumour, immunosuppressive, and so on (Reno et al., 2015; Hu et al., 2017; Chen et al., 2018; Lv et al., 2019). *Tripterygium* glycosides tablet (TGT), as the most prevalent preparation of TWHF, is frequently used in the treatment of autoimmune diseases, including nephrotic syndrome (Wu et al., 2022) and rheumatoid arthritis (Lin et al., 2021). With the expansion of clinical applications, increasing reports of adverse drug reactions caused by TGT have been reported. Severe hepatotoxicity (Guo et al., 2023), nephrotoxicity (Wang et al., 2018), reproductive toxicity (Ge et al., 2023), and cardiovascular system toxicity (Du et al., 2018) have been established, significantly restricting the clinical utilisation of TGT. Therefore, it is urgent to research the efficacy and toxicity material basis of TGT, which is significant to guide the formulation optimisation and quality control of TGT.

To date, over 100 chemical compounds have been identified from TGT, mostly with alkaloids, sesquiterpenes, diterpenes and triterpenes (Qu et al., 2015; Zhang et al., 2019). As outlined in Current National Drug Standards (WS3-B-3350-98-2011), each tablet of TGT shall contain no more than 10 µg of Triptolide and no less than 10 µg of wilforlide A (Zhang et al., 2020). Nevertheless, owing to the complexity of the components in TGT, the overall efficacy and toxicity of the preparation is not fully reflected by only two terpenoid components as quality control indicators. Our research group based on the ‘Prospective Observational Cohort Study on Factors Influencing the Prognosis of Chronic Kidney Disease Patients’ (Ethic NO. 2016-023-01) found that patients still suffered adverse reactions when taking TGT from some manufacturers while following the drug dosage. In our previous study, we found that the alkaloid content in TGT from various manufacturers exhibited significant variability (Gao et al., 2019), and a significant positive connection existed between alkaloid content and clinical adverse reactions. Therefore, we inferred that alkaloids of TGT may be important toxic components that cause adverse effects.

The characteristics of traditional Chinese medicines (TCMs) include systematism, wholeness, and multi-component synergy arising from their complex chemical constituents. The clarification of the material basis of efficacy and toxicity is the key issue for elucidating the overall efficacy and quality control of TCMs (Jiang et al., 2010). The constituents knock-out/knock-in technology takes the spectrum-effect relationship of TCMs as an entry point, which was derived from the knock-out/knock-in studies utilised in genetic research (Xu et al., 2023), and uses the techniques of systematic solvent extraction, column chromatography and preparative liquid chromatography to accurately and efficiently extract and isolate the target components (Cui et al., 2020). With the help of chromatographic fingerprints, especially HPLC fingerprints, the similarity between the chromatographic peaks was evaluated by comparing the changes in the fingerprints of the samples to identify the target components (Yang et al., 2022). To achieve the purpose of separating the target components from the medicine without loss or damage. Finally, the efficacy and toxicity changes of the target components before and after knockout/knock-in were compared by *in vivo*/*in vitro* experiments, and then the role of the target components in the medicine was studied (Jin et al., 2013).

In this study, constituents knock-out/knock-in technology was used to extract and enrich the alkaloidal components of TGT with the C_18_ column chromatography and chromatographic fingerprints. Biochemical indices and renal histopathology of adenine-induced CKD rats were analysed after the drug intervention for evaluating the efficacy and toxicity material basis of TGT. Non-target metabolomics of plasma was used to search for potential efficacy biomarkers, and 16S rRNA sequencing was applied to analyse variations in rat colonic microbiota to clarify the mechanism of TGT in the therapy of CKD. Finally, a UPLC-MS/MS approach was utilised for the quantitative analysis of relevant gut-derived uremic toxins and their precursors in plasma, urine, and faeces to study the mechanism of TGT in the treatment of CKD via the gut-liver-kidney axis. Overall, this study aimed to elucidate the efficacy and toxicity material basis and their mechanisms in the treatment of CKD with TGT and to provide a reference for the process optimisation and quality control of TGT.

## 2 Materials and methods

### 2.1 Reagents and drug

Triptonide, wilfortrine, wilfordine, wilforgine, wilforine, and celastrol were purchased from Chengdu DeSiTe Biotechnology Co., Ltd (Chengdu, China). Indoxyl sulfate (IS), *p*-cresyl sulfate (pCS), trimethylamine *N*-oxide (TMAO), and 3-fluoro-DL-valine were purchased from Sigma-Aldrich (St. Louis, United States of America). Tryptophan, tyrosine, choline, L-carnitine, and betaine were purchased from Beijing Solarbio Biotechnology Co., Ltd (Beijing, China). Hydrochlorothiazide was purchased from Shanghai Rhawn Chemical Technology Co., Ltd (Shanghai, China). Adenine was purchased from Shanghai Macklin Biochemical Technology Co., Ltd (Shanghai, China). The purity of all standards was above 98%. *Tripterygium* glycosides tablet (No. 2305108B) was purchased from Zhejiang DND Pharmaceutical Co., Ltd (Zhejiang, China). Chromatography-grade methanol and acetonitrile were purchased from Fisher Scientific (Fairlawn, United States of America). Deionised water was purchased from Guangzhou Watson’s Food and Beverage Co., Ltd (Guangzhou, China). Formic acid and ammonium formate were purchased from Shanghai Rhawn Chemical Technology Co., Ltd (Shanghai, China).

### 2.2 Extraction and isolation of AEF

The crushed TGT (450 g) was extracted using anhydrous ethanol (m/v, 100 g: 1 L) by ultrasonic extraction for 30 min, and the supernatant was filtered through filter paper to collect the filtrate. The residue was re-extracted until it became colourless. All collected filtrates were pooled and concentrated at reduced pressure, yielding 61.6 g of crude extract. Then, the extract of TGT (40 g) was distributed as ten fractions (Fr. 1 to Fr. 10) through C_18_ column (100 Å, 50 μm, QuikSep, China) with ethanol-water (1: 9→1: 4→3: 7→2: 3→1: 1→3: 2→7: 3→4: 1→9: 1→1: 0). The eluates were dried by rotary evaporation and lyophilised to obtain powders of ten fractions.

### 2.3 Qualitative analyses of AEP

#### 2.3.1 TLC analysis

The powders of ten fractions (Fr. 1 to Fr. 10) were solubilised in methanol to a concentration of 0.5 mg/mL 10 μL of each sample was spotted onto a high-silica gel GF pre-coated plate for TLC, which was unfolded with cyclohexane: acetone (5: 4), then removed and dried the plate. Finally, spray with Dragendorff’s reagent (Macklin, Shanghai, China) until the spots are clearly coloured, and examine in daylight.

#### 2.3.2 HPLC chromatographic fingerprints analysis

The extract of TGT and the powders of ten fractions (Fr. 1 to Fr. 10) were weighed precisely and solubilised in methanol to yield samples at a concentration of 0.5 mg/mL. The standards triptonide, wilfortrine, wilfordine, wilforgine, wilforine, and celastrol were weighed precisely and dissolved in methanol to yield samples at a concentration of 20 μg/mL.

The LC-20A chromatograph (Shimadzu, Japan) in conjunction with a diode array detector (DAD) source was used for fingerprints analysis. An UItimate AQ-C18 column (250 mm × 4.6 mm, 5 μm, Welch, China) was used for component separation. The mobile phase was 0.1% formic acid in water (phase A) and acetonitrile (phase B), and the flow rate was set at 1.0 mL/min. The column temperature was set at 30 °C, the DAD detection wavelength was 254 nm, and the injection volume was 10 μL. The specific gradient for phase B was as follows: 0 min: 15%; 15 min: 20%; 35 min: 25%; 60 min: 28%; 75 min: 32%; 100 min: 40%; 130 min: 45%; 145 min: 50%; 185 min: 60%; 205 min: 65%; 215 min: 90%; 230 min: 90%; 235 min: 15%; 240 min: stop.

The chromatographic peaks of the fingerprints were analysed by the Similarity Evaluation System for Chromatographic Fingerprint of TCM (Version 2012). The precision, stability, and repeatability were well validated.

### 2.4 Animals

Male Sprague-Dawley (SD) rats (180–200 g) were purchased by Guangzhou Ruige Biological Technology Co., Ltd (SCXK (Yue) 2021-0059, Guangdong, China). All rats were housed in a specific pathogen-free facility at Guangdong Pharmaceutical University (Guangdong, China). Light/dark cycle of 12/12 h, temperature was 20 °C–24 °C, and humidity was 40%–60%. The animal experiments adhered to the relevant regulations of the Experimental Animal Ethics Committee of Guangdong Pharmaceutical University (Ethic No. Gdpulacspf 2022439, approval date: 5 January 2024).

### 2.5 Animal studies

Male SD rats were randomly assigned to five groups, eight rats in each group, as follows: control group (Control), CKD model group (Model), TGT treatment group (TGT), alkaloid-enriched fraction treatment group (AEF), and non-alkaloid-enriched fraction treatment group (NAEF). Except for the control group, other SD rats were administered 200 mg/kg/day of adenine intragastrically for 3 weeks to establish the CKD model. Following the establishment of the model, the drug intervention was performed for 4 weeks. All animals were euthanised following anaesthesia with isoflurane. Blood, faeces, and kidneys were taken for subsequent analysis.

All rats were gavaged at a 10 mL/kg/day volume. The drug interventions were as follows: 1) Control and CKD model groups: intragastric administration of 0.5% CMC-Na; 2) TGT treatment group: intragastric administration of extract of TGT (10 mg/kg/day); 4) AEF treatment group: intragastric administration of alkaloid-enriched fraction (7.3 mg/kg/day); 5) NAEF treatment group: intragastric administration of the non-alkaloid-enriched fraction (2.7 mg/kg/day).

### 2.6 Biochemical index and histomorphology analysis of kidney

After the drug interventions were completed, 24-h urine and plasma samples were collected from rats. Creatinine, urea nitrogen, uric acid, urinary microalbumin, and cystatin C levels in plasma and urine were measured by the AU5800 fully automated biochemical analyser (Beckman Coulter, United States of America). Creatinine clearance (CrCl, Equation 1), cystatin C–based eGFR (eGFRcys, Equation 2), and kidney index (Equation 3) were calculated (Stevens et al., 2024; Ávila et al., 2025). The equations used were as follows:

$$CrCl=\left(UCr\times V\right)\;/\;PCr$$ (1)

where *CrCl* is the creatinine clearance (mL/min), *UCr* is the urinary creatinine concentration (μmol/L), *V* is the urine flow rate (mL/min), and *PCr* is the plasma creatinine concentration (μmol/L).

$$eGFRcys=133\times {\left(Pcys\;/\;0.8\right)}^{‐1.328}\times {\left(0.996\right)}^{age}\;\left(male\right)$$ (2)

where *Pcys* is the plasma cystatin C concentration (mg/L), *age* is age in years.

$$Kidney\;Index\;\left(\%\right)=\left(KW\;/\;BW\right)\times 100\%$$ (3)

where *KW* is the wet weight of the kidney (g) and *BW* is the total body weight (g) of the same individual.

The kidney tissue was dehydrated, encapsulated in paraffin, and sectioned. Tissue sections were stained with haematoxylin-eosin (H&E) or ponceau S acid fuchsin stain-aniline blue (Masson) and observed by GScan-60 fully automatic digital slide scanner (G cell technology, China). Finally, the collagen volume fraction (CVF) of the tissues in Masson sections was calculated by ImageJ.

### 2.7 Biochemical index and histomorphology analysis of kidney

#### 2.7.1 Preparation of plasma sample

50 μL of plasma samples were added to 300 μL of acetonitrile: methanol (1: 4, V/V) containing internal standards and vortexed for 3 min. After centrifugation at 12,000 rpm for 10 min at 4 °C, 200 μL of the supernatant was collected and then centrifuged at 12,000 rpm for 3 min at 4 °C. The supernatant was collected for LC-MS analysis. In addition, equal quantities of each sample were combined to produce quality control (QC) samples to evaluate instrument and metabolite stability.

#### 2.7.2 LC-MS condition

LC-30A chromatography (Shimadzu, Japan) in conjunction with Triple TOF™ 6600^+^ mass spectrometer (AB SCIEX, United States of America) was utilised for the quantification of plasma metabolites. The metabolites were separated using an ACQUITY Premier HSS T3 column (100 mm × 2.1 mm, 1.8 µm, Waters, United States of America). The mobile phase was 0.1% formic acid in water (phase A) and 0.1% formic acid in acetonitrile (phase B), and the flow rate was set at 0.4 mL/min. The column temperature was set at 40 °C, and the injection volume was 4 μL. The specific phase B gradient was as follows: 0 min: 5%; 2 min: 20%; 5 min: 60%; 6 min: 99%; 7.5 min: 99%; 7.6 min: 5%; 10 min: stop.

Electrospray ionisation (ESI) source was utilised, with the precursor ion mass scan range was 50–1,000 Da and the product ion mass scan range was 25–1,000 Da. The source parameters were set as follows: GS1: 50 psi; GS2: 50 psi; CUR: 25 psi; TEM: 550 °C; declustering potential: 60 V or −60 V, respectively; ion spray voltage floating: 5000 V or −4000 V, respectively.

### 2.8 16S rRNA detection of faecal microbiome

After the drug interventions were completed, the colonic contents of the rats were collected. Total microbial DNA was collected from faecal samples utilising the CTAB method, and the concentration and purity of the DNA were assessed by 1% agarose gels. DNA was diluted to a concentration of 1 ng/μL using sterile water. The V3-V4 region of the microbial 16S rRNA gene was amplified utilising the primer pair 341F (5′-CCTAYGGGRBGCASCAG-3′) and 806R (5′-GGACTACNNGGGGTATCTAAT-3′). PCR products were extracted from 2% agarose gels and purified with the Qiagen Gel Extraction Kit (Qiagen, Germany). Purified amplicons were sequenced with the Illumina NovaSeq platform to assess the diversity of gut bacteria in faecal samples.

### 2.9 LC-MS/MS analysis of uremic toxins

We developed two methods for quantification of uremic toxins and related metabolites due to their complexity and inability to complete the separation on the same column. The specificity, linearity, intra- and inter-day accuracy and precision, recovery, and stability were thoroughly validated, as detailed in our prior study.

#### 2.9.1 The LC-MS/MS method for C_18_ column

The LC-MS/MS 8045 mass spectrometer (Shimadzu, Japan) was employed for the analysis of metabolites associated with uremic toxins. The separation of metabolites was performed by an ACQUITY UPLC BEH C_18_ (50 mm × 2.1 mm, 1.7 µm, Waters, United States of America). The mobile phase was 0.1% formic acid in water (phase A) and acetonitrile (phase B), and the flow rate was set at 0.3 mL/min. The column temperature was set at 45 °C, and the injection volume was 1 μL. The specific gradient for phase B was as follows: 0 min: 15%; 0.5 min: 15%; 2 min: 20%; 2.5 min: 85%; 3 min: 85%; 3.5 min: 15%; 10 min: stop.

Electrospray ionisation (ESI) source was utilised, and the source parameters were set as follows: interface temperature: 300 °C; heating gas flow: 10 L/min; nebulising gas flow: 3 L/min; DL temperature: 250 °C; drying gas flow: 10 L/min; heating block temperature: 400 °C. MRM method parameter was set as Supplementary Table S1.

#### 2.9.2 The LC-MS/MS method for hilic column

The LC-MS/MS 8045 mass spectrometer (Shimadzu, Japan) was employed for the analysis of metabolites associated with uremic toxins. The separation of metabolites was performed by an ACQUITY UPLC BEH HILIC (100 mm × 2.1 mm, 1.7 µm, Waters, United States of America). The mobile phase was 10 mM ammonium formate in water (phase A) and 0.1% formic acid in acetonitrile (phase B), and the flow rate was set at 0.3 mL/min. The column temperature was set at 45 °C, and the injection volume was 1 μL. The specific gradient for phase B was as follows: 0 min: 75%; 1 min: 75%; 2 min: 70%; 2.5 min: 70%; 3 min: 50%; 4 min: 50%; 4.5 min: 75%; 11 min: stop.

The mass spectrometry parameter settings remain identical to those in Section 2.9.1., and the MRM method parameter was set as in Supplementary Table S1.

### 2.10 LC-MS/MS analysis of uremic toxins

50 μL of plasma or urine samples were combined with 50 μL of acetonitrile containing 3.2 μg/mL internal standard, followed by the addition of 100 μL of acetonitrile. After vortexing for 30 s, it was left at 4 °C for 1 h to extract metabolites and precipitate proteins. The samples were centrifuged at 12,000 rpm for 5 min at 4 °C. 50 μL of the supernatant was extracted, to which 50 μL of deionised water was added and vortexed for 30 s for LC-MS analysis. Fresh faeces were combined with 3 times their volume of deionised water, mixed to create a homogenate, then centrifuged at 12,000 rpm for 5 min at 4 °C. 50 μL of the supernatant were collected and then combined with 50 μL of acetonitrile containing 3.2 μg/mL internal standard, followed by the addition of 100 μL of acetonitrile. After vortexed for 30 s and left at 4 °C for 1 h, the samples were centrifuged at 12,000 rpm for 5 min at 4 °C. 50 μL of the supernatant was collected, followed by the addition of 50 μL of deionised water, and the mixture was vortexed for 30 s for LC-MS analysis.

### 2.11 Statistical analysis

All data were analysed using GraphPad Prism 9.5.1. One-way ANOVA was used for statistical comparisons and was presented as mean ± standard deviation (SD). *P* < 0.05 was considered statistically significant (^*^ *p* < 0.05, ^**^ *p* < 0.01, ^***^ *p* < 0.001, ^****^ *p* < 0.0001).

## 3 Results

### 3.1 Outline of TGT chemistry and enrichment of AEF and NAEF

**FIGURE 1:**
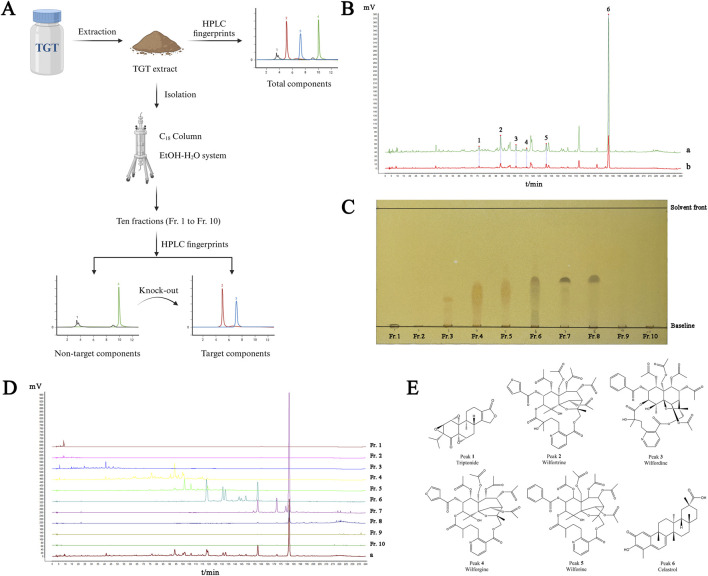
The fingerprint of TGT and the enrichment of AEF and NAEF **(A)** Schematic diagram of the constituents knock-out/knock-in experiment **(B)** The fingerprint of TGT (a) and mixture of ten fractions (b). **(C)** The TLC of ten fractions **(D)** The fingerprint of TGT **(A)** and ten fractions **(E)** Chemical structures of standards.

In this study, the qualitative method of TLC and HPLC fingerprinting for TGT was established based on the constituents knock-out/knock-in technology for accurately reflecting the outline of TGT chemistry and enrichment of AEF and NAEF. The experimental scheme was shown in Figure 1A. The corresponding methodological validation of HPLC fingerprinting was carried out (Supplementary Tables S2–S4). The extract of TGT was distributed by C_18_ column, and ten fractions (Fr. 1 to Fr. 10) were obtained. Through comparing the HPLC fingerprinting of each fraction (Figure 1D), it was found that the constituents among each fraction were clearly segmented, although there was slight crossover between each fraction. The overall retention of the compound composition was good, indicating no significant loss of components during the separation.

By comparing the retention time and UV spectrum of fingerprint peaks from the standard and the sample (Figure 1B), it was determined that peaks 1-6 were respectively triptonide, wilfortrine, wilfordine, wilforgine, wilforine, and celastrol (Figure 1E). It was found that the characteristic components of TGT, sesquiterpene pyridine alkaloids, were mainly concentrated between 90 min and 135 min. Through comparing the chromatograms of each fraction, it was found that the sesquiterpene pyridine alkaloids were mainly concentrated in Fr. 4 to Fr. 6. And the alkaloidal components were found to be concentrated in Fr. 3 to Fr. 6 by TLC and Dragendorff’s reagent colour (Figure 1C).

In summary, Fr. 3 to Fr. 6 were combined as AEF, and the remaining fractions were combined as NAEF. The weights of AEF and NAEF were, respectively, 27.34 g and 9.72 g, with the ratio of the two approximately 2.8:1. According to the clinical dose, the oral dose of TGT for rats was 10 mg/kg/day, so at the same clinical dose, the oral doses of AEF and NAEF were 7.3 and 2.7 mg/kg/day, respectively.

### 3.2 TGT improved adenine-induced CKD in rats

**FIGURE 2:**
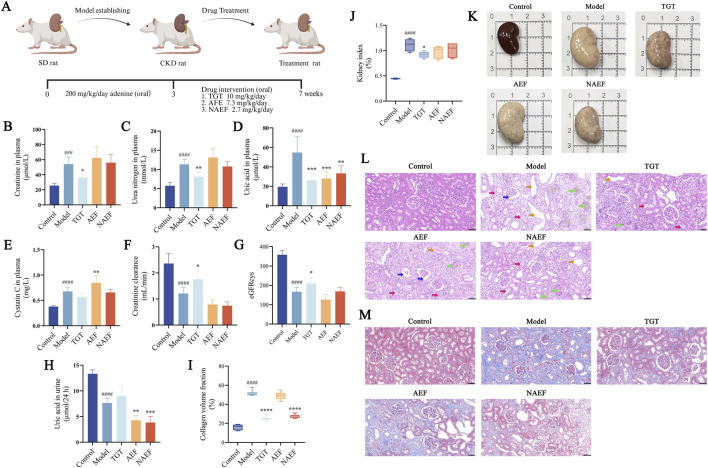
TGT ameliorated adenine-induced CKD rat model **(A)** Experimental scheme. The levels of plasma creatinine **(B)**, urea nitrogen **(C)**, uric acid **(D)** and cystatin C **(E,F)** Creatinine clearance **(G)** Cystatin C–based estimated glomerular filtration rate **(H)** The 24 h cumulative excretion of uric acid in urine **(I)** Renal collagen volume fraction **(J)** Kidney index. The data are shown as mean ± SD, compared with control group, ^###^ *p* < 0.001, ^####^ *p* < 0.0001; compared with model group, ^*^ *p* < 0.05, ^**^ *p* < 0.01, ^***^ *p* < 0.001, ^****^ *p* < 0.0001, *n* = 6 **(K)** Kidney appearance **(L)** H&E staining results of kidneys **(M)** Masson staining results of kidneys. The scale bar was 50 μm.

The experimental design was illustrated in Figure 2A. During the drug interventions, one rat died in the AEF group and none died in the other groups. The plasma of the dead rat was measured and found that creatinine, urea nitrogen, and cystatin C were as high as 259 μmol/L, 50.64 mmol/L, and 3.14 mg/L, respectively. The modelling results are shown in Figures 2B,C. The levels of creatinine (*p* < 0.001) and urea nitrogen (*p* < 0.0001) in the model group significantly increased compared to the control group, indicating that the CKD model was established and could be sustained during the experiment.

Following 4 weeks of TGT treatment, the plasma creatinine level was significantly declined (*p* < 0.05) compared with the model group (Figure 2B). The plasma creatinine level in the AEF group was increased by 15.1% in comparison to the model group. No significant difference was found between the NAEF group and the model group. In Figure 2C, the plasma urea nitrogen level in the TGT group was significantly reduced compared to the model group (*p* < 0.01), whereas the NAEF group showed a slight, non-significant decrease of 4.8% in plasma urea nitrogen level. But the plasma urea nitrogen level in the AEF group was higher by 15.9% with no significant difference compared to model group. As shown in Figure 2D, the plasma uric acid levels in the TGT group, AEF group, and NAEF group decreased significantly by 52.1% (*p* < 0.001), 48.8% (*p* < 0.001), and 39.1% (*p* < 0.01), respectively, in comparison to the model group. Cystatin C, as an endogenous marker reflecting glomerular filtration function (Haines et al., 2023), was measured in the plasma level for evaluation of renal function in rats. In Figure 2E. The cystatin C levels in the TGT and NAEF groups decreased by 15.9% and 3.1%, respectively, compared to the model group, without significance. The renal impairment was more severe in the AEF group, with cystatin C level significantly increased (raised by 25.1%, *p* < 0.01) in comparison to the model group.

Next, the 24-h cumulative excretion of creatinine and uric acid in urine samples was measured for better observing the efficacy of drug intervention. As shown in Supplementary Figure S1. After modelling, the excretion of creatinine in model group was reduced by 5.4%, without significance in comparison to the control group. The creatinine excretion level was significantly decreased in the NAEF group (*p* < 0.0001), whereas the AEF group showed a non-significant reduction of 1.7% in comparison to the model group. But the excretion of creatinine in TGT group was increased by 7.3% with no significant difference compared to model group. To determine renal function, CrCl was calculated for each group. As shown in Figure 2F, the CrCl of the model group was significantly reduced (*p* < 0.0001) in comparison to the control group. After drug interventions, the CrCl was increased significantly in the TGT group (*p* < 0.05) in comparison to the model. The CrCl of the AEF and NAEF groups decreased by 34.5% and 38.6%, respectively, compared to the model group, with no significance. Considering that in CKD, residual nephrons substantially enhance creatinine secretion. Meanwhile, AEF may competitively inhibit creatinine transporters in renal proximal tubules (OCT2 and MATE), thereby interfering with the tubular secretion of creatinine and increasing its concentrations in the plasma (Ávila et al., 2025). Under these conditions, creatinine clearance tends to be underestimated. In contrast, cystatin C is exclusively filtered by the glomerulus and undergoes minimal tubular secretion. Therefore, we further assessed estimated GFR based on measurements of cystatin C (eGFRcys). In Figure 2G, the eGFRcys of the model group significantly decreased compared to the control group (*p* < 0.0001). Similar to CrCl, the eGFRcys in the TGT group showed a significant increase (*p* < 0.05) when compared to the model group. The eGFRcys of the AEF group decreased by 24.4% compared with the model group, without statistical significance. In Figure 2H, compared to model group, the excretion of uric acid in TGT group was increased with no significantly by 17.4%. The excretion of uric acid in the AEF and the NAEF groups decreased significantly by 44.1% (*p* < 0.01) and 49.8% (*p* < 0.001), respectively, in comparison to the model group.

As shown in Figure 2J, kidney index was decreased significantly in TGT group compared with model group (*p* < 0.05). The kidney appearance was shown in Figure 2K. Compared to the control group, the kidney in the model group became larger in size and greyish in colour. After drug interventions, the kidneys improved to a slight brick red colour and decreased in volume in the TGT and NAEF groups, but there was no change in the AEF group. The H&E results are shown in Figure 2L, the model group showed glomerular atrophy with enlarged cystic cavity (blue arrow), large amounts of renal calcium salt precipitation (green arrow), tubular vacuolar degeneration (red arrow), and tubular lumen dilatation (orange arrow). After the TGT and NAEF interventions, the glomerular structure was improved, the number of normal renal tubules was increased, and renal calcium salt precipitation was decreased. In the AEF group, there was obvious tubular vacuolar degeneration, renal calcium salt precipitation, and also destruction of the glomerular structure. The degree of renal fibrosis was evaluated through measuring the CVF of tissues in Masson staining (Figure 2M) by ImageJ. It was found that the renal CVF was significantly reduced after TGT and NAEF interventions (*p* < 0.0001), compared with control group (Figure 2I).

### 3.3 TGT modified the plasma metabolite composition in rats with CKD

**FIGURE 3:**
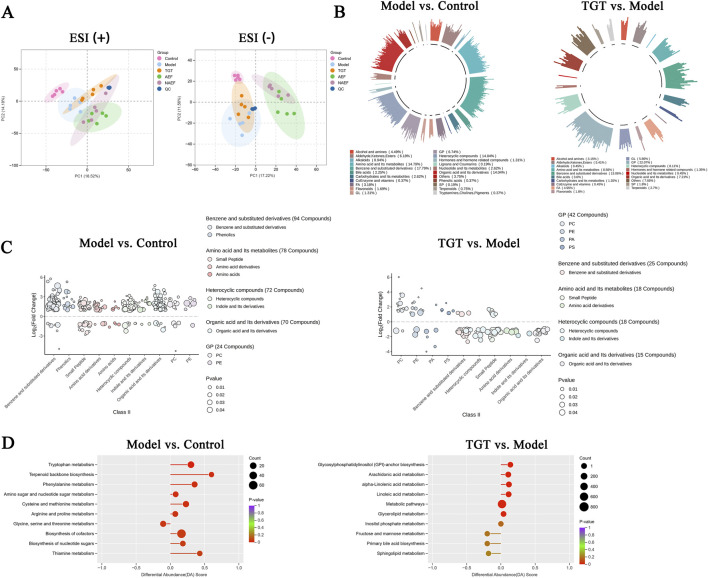
TGT altered the composition of the plasma metabolites in CKD rats **(A)** PCA plot in both positive and negative modes **(B)** The primary classification of differential metabolites in model and TGT groups **(C)** The top five differential metabolites in model and TGT groups **(D)** Analysis of pathways with different metabolites in model and TGT groups.

We conducted a non-targeted metabolomics study of plasma to examine the compositional changes in plasma metabolites and assess the impact of drug interventions on these metabolites. The PCA model was used to evaluate the similarity among the samples, revealing a realistic distribution. As shown in Figure 3A, the QC samples exhibited good aggregation, indicating steady analytical conditions throughout the process. The metabolic profiles of the TGT group were overlapped with the model group but closed to the control group, showing that the metabolites of CKD rats could be effectively improved after TGT intervention. However, the AEF and NAEF groups had a distinct trend of divergence from the other groups, indicating that AEF and NAEF considerably altered the metabolites. The OPLS-DA model was shown in Supplementary Figure S2, with *R* ^2^ Y and Q^2^ values >0.5 and a *P* value <0.05, indicating that the OPLS-DA model has good interpretative and predictive abilities. Metabolites that satisfied the conditions of VIP > 1, *P* value <0.05, FC ≥ 2, or FC ≤ 0.5 were classified as differential metabolites and represented in the volcano map. As shown in Supplementary Figure S3, A total of 534 differential metabolites were identified in the model group compared to the control group, while 222, 461, and 471 differential metabolites were observed in the TGT, AEF, and NAEF groups compared to the model group, respectively.

We studied the primary classification of differential metabolites in each group to detect the alterations in plasma metabolites of rats. As shown in Figure 3B, amino acids and their metabolites, indole and its derivatives, organic acids and their derivatives, and heterocyclic compounds occupied the large majority of the metabolites after modelling. After drug interventions (Figure 3B; Supplementary Figure S4), the TGT group had significantly decreased amounts of the above four metabolite classes and an improved amount of glycerophospholipids (GPs) compared to the AEF and NAEF groups. To identify the changes in metabolites, we analysed the top five differential metabolites in each group. As shown in Figure 3C, a large number of metabolite levels were significantly upregulated after modelling, especially benzene and substituted derivatives, indole and its derivatives, and organic acids and their derivatives. After TGT intervention (Figure 3C), metabolite levels were significantly downregulated, especially amino acid derivatives, indole and its derivatives, and organic acid and its derivatives were all downregulated, but levels of phosphatidylethanolamine (PE) and phosphatidylcholine (PC) were significantly upregulated. Notably, as shown in Supplementary Figure S5, the AEF and NAEF groups showed non-significant downregulation of the levels of various metabolites compared to the model group.

Next, metabolic pathway analysis was performed for the differential metabolites in each group. Tryptophan metabolism, phenylalanine metabolism, cysteine and methionine metabolism were significantly upregulated, while glycine, serine and threonine metabolism were significantly downregulated in the model group compared to the control group (Figure 3D). After drug interventions, the TGT group significantly affected glycosylphosphatidylinositol (GPI)-anchor biosynthesis and arachidonic acid metabolism (Figure 3D). However, after AEF and NAEF interventions (Supplementary Figure S6), folate biosynthesis was significantly decreased. It was reported that folic acid had a significantly renoprotective effect, and moderately supplemented folic acid could effectively slow down the progression of CKD and reduce the risk of CKD to uremia (Xu et al., 2016). We can infer that TGT could reduce the levels of benzene and substituted derivatives, indole and its derivatives, and organic acid and its derivatives, and increase the level of glycerophospholipids, which may enhance the efficiency of TGT in renal protection.

### 3.4 TGT modifies the gut microbiota in rats with CKD

**FIGURE 4:**
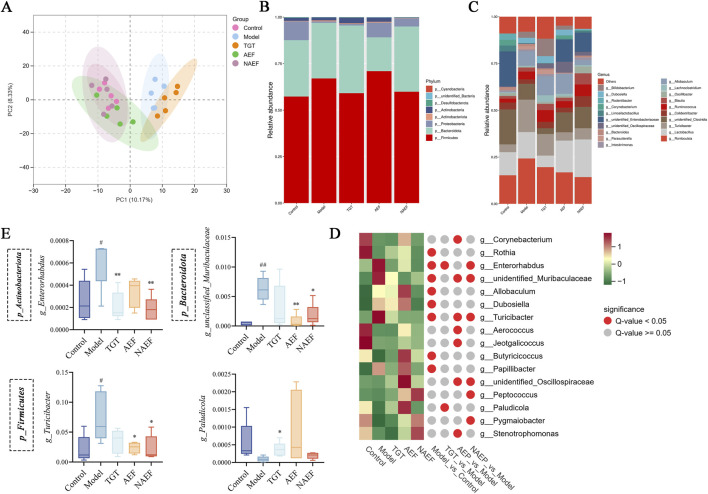
TGT modulated the diversity of gut microbiota in CKD rats **(A)** PCA plot of each group. The relative abundance at the **(B)** phylum level and **(C)** genus level **(D)** Heatmap of the gut microbiota at the genus level **(E)** Box plot comparison of the differential microbiota among the treatment groups. Compared with control group, ^#^ *p* < 0.05, ^##^ *p* < 0.01; compared with model group, ^*^ *p* < 0.05, ^**^ *p* < 0.01, *n* = 5.

We performed 16S rRNA analysis to study the compositional alterations of the gut microbiota in CKD and the impact of TGT on the intestinal flora in CKD rats. The Chao one index, Simpson index, and Shannon index are commonly utilised indicators for *α*-diversity. In Supplementary Figure S7, *α*-diversity was not significantly reduced in the model group, indicating that the microbial species diversity of the intestinal microbiota was not significantly disrupted. According to the PCA result in Figure 4A, the AEF and NAEF groups showed overlap with the control group, indicating that the interventions improved the abnormalities in the gut bacteria of CKD rats. Finally, the TGT group overlapped with the model group and showed more deviation from the control group, suggesting the distinct and intricate capabilities of TGT to regulate gut microbiota, maybe associated with its therapeutic mechanism for CKD.

Compared to the model group, the abundance of *p_Firmicutes* was reduced to the level of the control group after TGT and NAEF interventions. Notably, after the AEF intervention, the abundance of *p_Firmicutes* was not altered, but the abundance of *p_Bacteroidota* was clearly reduced, compared with the model group. We analysed all 63 identified bacterial genera (Figure 4C), selected the differential genera, and displayed them as a heat map (Figure 4D). Compared to the control group, it can be seen that *g_Enterorhabdus*, *g_unidentified_*Muribaculaceae, *g_Allobaculum*, *g_Dubosiella*, *g_Turicibacter*, and *g_Papillibacter* showed significant enrichment in the model group, while *g_Rothia* and *g_Butyricicoccus* showed significant depletion in the model group. After drug interventions, we found some significant changes in bacterial genera that were closely related to the progression of CKD. *g_Enterorhabdus* has been reported to have the capability to generate short-chain fatty acids (SCFAs), such as butyric acid (Morrison and Preston, 2016; Ren et al., 2023), which has renoprotective effects (Li et al., 2019) and can affect the gut-kidney axis through a variety of mechanisms, thereby improving CKD progression (Stanford et al., 2020). *G_Turicibacter* has been reported to have a positive correlation with serum levels of indole-3-acetic acid and indole-3-propionic acid (Xie et al., 2024), which are metabolised from tryptophan and belong to the indole class of uremic toxins (Lauriola et al., 2023). *G_unclassified_*Muribaculaceae, a member of *p_Bacteroidota*, has been identified to contain genes that encode oxalate-degrading enzymes, which could have a renoprotective effect by decreasing urinary oxalate excretion and calcium oxalate crystal deposition of the kidneys, facilitated by enhanced intestinal oxalate degradation (Wang Y. et al., 2023). *G_Paludicola* has been reported to correlate positively with choline TMA-lyase (CutC) activity, and choline is converted to trimethylamine (TMA) by CutC, then it is oxidised to TMAO by flavin-containing monooxygenases (FMOs) (Guo et al., 2024), TMAO is a typical uremic toxin (Ji et al., 2021). In Figure 4E, the abundance of *g_Enterorhabdus* (*P* < 0.01) and *g_Paludicola* (*P* < 0.05) was significantly regulated after TGT intervention, compared to the model group. After AEF and NAEF interventions, the abundance of *g_Turicibacter* (*P* < 0.05 and *P* < 0.05, respectively) and *g_unclassified_*Muribaculaceae (*P* < 0.01 and *P* < 0.05, respectively) was significantly regulated in comparison to the model group. We could assume that TGT may modulate the abundance of intestinal flora that produces uremic toxins, thereby reducing uremic toxins in the body and promoting the production of SCFAs, potentially improving the renal protective efficacy of TGT. However, the difference in the bacterial regulatory ability of the drugs resulted in the AEF and NAEF interventions not showing the same therapeutic effect as TGT.

### 3.5 TGT reduced *in vivo* levels of gut-derived uremic toxins

With the help of non-target metabolomics and 16S rRNA analysis, we inferred that TGT can exert a renoprotective function by improving amino acid metabolism disorders and dysbiosis of intestinal flora to regulate the gut-derived uremic toxins *in vivo*. It was necessary to evaluate the gut-derived uremic toxins regulatory ability of TGT. The LC-MS/MS methods for analysis of uremic toxins and their precursors in plasma, faecal, and urine samples were established. All analytical methods have undergone methodological validation (Supplementary Tables S5–S10).

**FIGURE 5:**
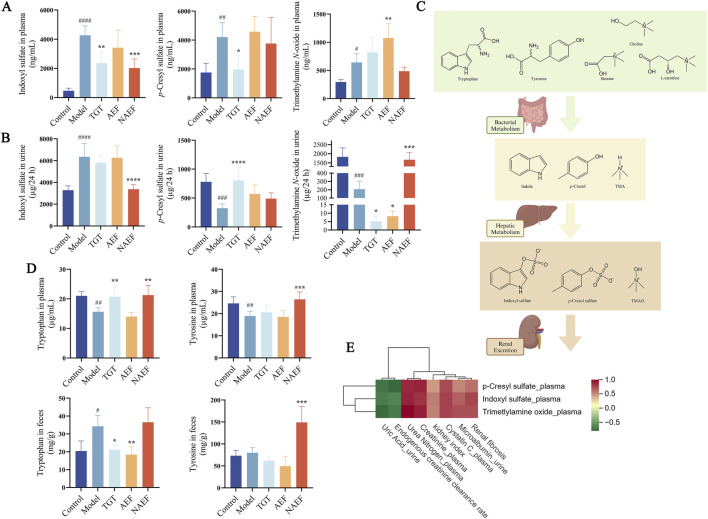
TGT modulated the gut-derived uremic toxin levels in CKD rats **(A)** The content of indoxyl sulfate, *p*-cresyl sulfate, and trimethylamine *N*-oxide in plasma **(B)** The content of indoxyl sulfate, *p*-cresyl sulfate, and trimethylamine *N*-oxide in urine **(C)** Schematic diagram of the gut-derived uremic toxins production pathway **(D)** The content of tryptophan and tyrosine in plasma and faeces **(E)** Heatmap of the correlation analysis between plasma uremic toxins and renal function indicators. The data are shown as mean ± SD, compared with control group, ^#^ *p* < 0.05, ^##^ *p* < 0.01, ^###^ *p* < 0.001, ^####^ *p* < 0.0001; compared with model group, ^*^ *p* < 0.05, ^**^ *p* < 0.01, ^***^ *p* < 0.001, ^****^ *p* < 0.0001, *n* = 6.

After 4 weeks of pharmacological interventions, levels of IS, pCS, and TMAO were measured in plasma samples. As shown in Figure 5A, compared with the control group, the plasma levels of IS (*p* < 0.0001), pCS (*p* < 0.01), and TMAO (*p* < 0.05) were increased significantly in the model group. It was confirmed that with the development of CKD, the decline in the capacity of renal filtration and the dysbiosis of the intestinal flora result in the accumulation of uremic toxins *in vivo*, which was consistent with the previous report (Bain et al., 2006; Fujii et al., 2018). In Figure 5A, the plasma IS level significantly decreased in the TGT group (*p* < 0.01) and the NAEF group (*p* < 0.001) compared to the model group, although the plasma IS level in the AEF group also decreased, but not significantly (reduced by 19.9%). As shown in Figure 5A, in comparison to the model group, the plasma pCS level significantly decreased in the TGT group (*p* < 0.05), and the plasma pCS level in the NAEF group showed a non-significant reduction of 10.5%. However, the plasma pCS level in the AEF group showed a slight increase of 8.8%, with no significant difference compared to the model group. Notably, after AEF and TGT interventions (Figure 5A), the plasma TMAO level was increased significantly in the AEF group (*p* < 0.01), while the plasma TMAO level in TGT group was increased with no significantly (increased by 28.1%), compared with model group. But in NAEF group, the plasma TMAO level was reduced with no significantly by 24.1%, compared to model group. To identify the relationship between uremic toxins and CKD progression, we analysed the relationship between plasma uremic toxin levels and renal biochemical indices. As shown in Figure 5E, IS, pCS, and TMAO were significantly positively correlated with creatinine, urea nitrogen, renal fibrosis, and cystatin C, and significantly negatively correlated with Ccr and excretion of uric acid. It indicated that the *in vivo* level of uremic toxins is significantly related to the CKD progression and is an important indicator for the CKD.

Next, the 24 h cumulative excretion of IS, pCS, and TMAO in urine samples was measured for better observing the efficacy of drugs on renal filtration capacity. In Figure 5B, compared to the control group, the excretion of pCS (*p* < 0.001) and TMAO (*p* < 0.0001) in urine was reduced significantly in the model group, except for the excretion of IS in urine (*p* < 0.0001) was increased significantly. After drug interventions (Figure 5B), compared to the model group, the excretion of IS in urine declined significantly in the NAEF group (*p* < 0.0001), while the urinary excretion of IS in the TGT and AEF groups decreased slightly with no significant change by 8.5% and 1.2%, respectively. And the excretion of pCS in urine was increased significantly in the TGT group (*p* < 0.0001), while the excretion of pCS in urine in the AEF group and the NAEF group was increased with no significantly by 76.3% and 50.9%, respectively, compared to the model group. As shown in Figure 5B, the excretion of TMAO in urine was increased significantly in the NAEF group (*p* < 0.0001), compared with model group. The excretion of TMAO in the TGT and AEF groups decreased by 97.4% and 96.0%, respectively, compared to the model group, although this reduction was not significant.

Since gut-derived uremic toxins are produced from proteins through intestinal microbial fermentation and hepatic metabolism, the generation pathway is illustrated in Figure 5C. It is important to focus on the precursors of gut-derived uremic toxins. Tryptophan, tyrosine, choline, L-carnitine, and betaine levels were measured in plasma and faeces. The results are shown in Figure 5D and Supplementary Figure S8, compared to the control group, the plasma levels of tryptophan (*p* < 0.01), tyrosine (*p* < 0.01), and L-carnitine (*p* < 0.0001) were reduced significantly in the model group. We inferred that alterations in the intestinal microbiota of the CKD rats resulted in enhanced utilisation of tryptophan, tyrosine, and L-carnitine, which correlated with changes in plasma levels of IS, pCS, and TMAO. After the drug interventions, plasma tryptophan levels (Figure 5D) were significantly elevated in the TGT group (*p* < 0.01) and the NAEF group (*p* < 0.01), which was consistent with the trend of IS in plasma. But in AEF group, the plasma tryptophan level was reduced with no significantly by 10.4%, compared to model group. As shown in Figure 5D, compared with the model group, the plasma tyrosine level was increased significantly in the NAEF group (*p* < 0.05), while the plasma tyrosine level in TGT group was increased not significantly (increased by 8.7%). In Supplementary Figure S8, although plasma levels of L-carnitine (reduced by 65.2%, *p* < 0.0001), choline (increased by 33.7%, *p* < 0.01), and betaine (increased by 68.4%, *p* < 0.0001) were significantly changed in the model group compared with control group, there was no significant difference between the administered groups and the model group. Further focus on the precursors of gut-derived uremic toxins in faeces. In Figure 5D and Supplementary Figure S9, it can be seen that the levels of tryptophan (*p* < 0.05) and choline (*p* < 0.0001) of the faeces were significantly increased in the model group, whereas the levels of L-carnitine (*p* < 0.0001) and betaine (*p* < 0.01) of the faeces were significantly reduced in the model group. Compared to the model group, the faecal tryptophan levels were significantly reduced in the TGT (*p* < 0.05) and AEF (*p* < 0.01) groups (Figure 5D). As shown in Supplementary Figure S9, compared with the model group, the levels of L-carnitine (*p* < 0.01) and betaine (*p* < 0.0001) of the faeces were significantly increased in the AEF group, whereas the levels of choline (*p* < 0.01) of the faeces were significantly reduced in the AEF group. And there was no significant difference between the administered groups and the model group. This may be related to the significant increase in plasma TMAO levels after the AEF intervention.

In summary, TGT could significantly reduce plasma IS levels and regulate tryptophan back to normal levels *in vivo*. Meanwhile, non-target metabolomics of plasma revealed that TGT could reduce the levels of indole and its derivatives *in vivo*. Therefore, we inferred that TGT can regulate the tryptophan-indole-IS production pathway, thus decreasing the plasma concentration of IS. For pCS, TGT improved renal excretion of it, thereby reducing plasma pCS level. Notably, plasma levels and urinary excretion of three uremic toxins did not improve after AEF intervention, and even significantly increased plasma TMAO level (*p* < 0.01). Therefore, we inferred that AEF may be the important compound resulting in the toxic side effects of TGT.

### 3.6 Analysis of the relationship between uremic toxins and intestinal microbiota

**FIGURE 6:**
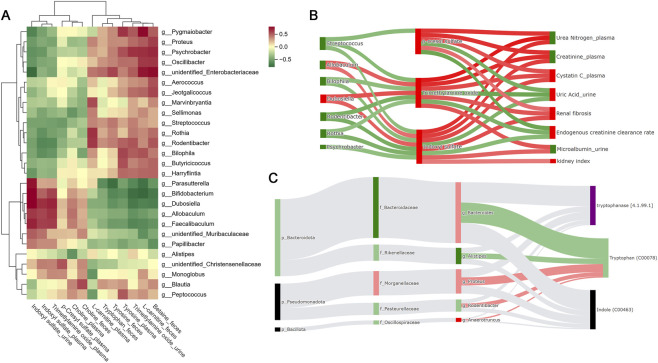
The relationship between uremic toxins and intestinal microbiota in CKD rats **(A)** Heatmap of the correlation study between uremic toxins and intestinal bacteria **(B)** Sankey analysis among uremic toxins, phenotype parameters, and gut bacteria **(C)** Sankey analysis of tryptophan metabolic pathway and gut bacteria of TGT vs. model group.

Since the TGT exhibited a distinctive microbial profile and the capacity to modulate the production of uremic toxins, it is essential to explain the correlation between intestinal microbiota and uremic toxins during the treatment of CKD with TGT. The correlation between gut microbiota and uremic toxins was evaluated using Spearman’s analysis, as shown in Figure 6A. It was shown that *g_Bifidobacterium*, *g_Dubosiella*, *g_Allobaculum*, and *g_Faecalibaculum* were positively correlated with IS, TMAO, and pCS. To better elucidate the biological and statistical correlations among uremic toxins, phenotype parameters, and gut bacteria, Sankey analysis was performed. *G_Allobaculum* and *g_Dubosiella* showed significant positive correlation with plasma IS and plasma TMAO levels. *G_Bilophila*, *g_Rodentibacter*, and *g_Rothia* showed a significant negative correlation with plasma IS and plasma TMAO levels. All of the above genera were significantly correlated with the urinary excretion of IS and TMAO and the levels of their precursors (Figure 6A). Notably, *g_Dubosiella*, *g_Allobaculum*, and *g_Faecalibaculum* were reported to be closely related to the synthesis of SCFAs, especially butyric acid (Tian et al., 2021; Huang et al., 2023; Chen et al., 2024), while butyric acid had a nephroprotective effect in CKD rats (Li et al., 2019). *G_Bilophila* had genomic markers indicative of genetic code extension, potentially allowing it to metabolise both TMA and its precursor chemicals, which would otherwise be utilised in host hepatic activities, therefore diminishing or bypassing TMAO synthesis (Kivenson et al., 2020).

Since TGT showed a significant inhibitory effect on uremic toxins, particularly the tryptophan-indole-IS production pathway. Next, we focused on the intestinal flora associated with the tryptophan metabolic pathway, as shown in Figure 6C. The widths of the bands are linearly proportional to the number of bacteria. The red or green colour of bars and bands indicates up- or downregulation of bacteria and metabolites, or positive or negative correlations between them. Compared to the model group, TGT significantly changed the abundance of *g_Bacteroides*, *g_Alistipes*, *g_Proteus*, *g_Rodentibacter*, and *g_Anaerotruncus*, which are closely related to tryptophanase, tryptophan, and indole. Notably, after TGT intervention, the abundance of *g_Bacteroides* was upregulated and the abundance of *g_Rodentibacter* was downregulated, which belong to the genera *f_*Bacteroidaceae and *f_*Pasteurellaceae, respectively, which encode tryptophan indole-lyase (TIL), mediating the degradation of tryptophan (Harris and Phillips, 2013; Devlin et al., 2016). We can infer that TGT affects the expression of TIL through regulating the abundance of *g_Bacteroides* and *g_Rodentibacter* and thus reduces indole production, which ultimately decreases IS level *in vivo*.

## 4 Discussion

CKD, as a progressive and irreversible loss of kidney function, has no effective cure or preventive strategies (Stevens et al., 2024). Since kidney function progressively diminishes, the retention of numerous solutes that would typically be discharged by the kidneys occurs, with those solutes that adversely affect biological functions referred to as uremic toxins. In recent years, the European Uremic Toxin Work Group on Uremic Toxins has proposed a classification of more than 100 uremic toxins (Duranton et al., 2012). Many of these uremic toxins originate from the metabolic processes of primary dietary components by gut microbiota (Vaziri et al., 2013). Dysbiosis of the gut microbiome plays an important role in many chronic diseases. Recent years have clarified the gut-kidney axis, revealing more relationships between gut bacteria metabolites and renal function (Yang et al., 2018). An increasing amount of research indicates that the interaction between host and microbiota is pathophysiologically relevant for patients with CKD. Therefore, renal function in CKD patients can be enhanced through modulating the gut microbes. In this study, TGT was split into AEF and NAEF with the help of C_18_ column and HPLC fingerprinting based on the constituents knock-out/knock-in technology. The adenine-induced CKD rat model was established to study the efficacy mechanism and toxic components of TGT in the treatment of CKD. We found that TGT significantly reduced plasma levels of creatinine, urea nitrogen, and uric acid, markedly ameliorated glomerular filtration function and renal histomorphology, attenuated kidney fibrosis, and improved the progression of CKD in rats. In contrast, AEF intervention increased plasma levels of creatinine, urea nitrogen, and cystatin C, and further impaired renal function. These findings indicate that AEF may be an important toxic fraction of TGT, contributing to its clinical adverse effects.

The gut-kidney axis can be categorised into metabolism-dependent and immunological pathways (Evenepoel et al., 2016). The metabolism-dependent pathway is chiefly facilitated by metabolites generated by the intestinal bacteria, which possess the ability to modulate host physiological activities. The interactions are bidirectional, CKD influences both the composition and metabolism of the gut microbiota, whereas some uremic toxins arise from microbial metabolism. Most of the protein-derived gut-produced uremic toxins are the gut microbiota products that degrade from aromatic amino acids, including indole (mainly generated from tryptophan), *p*-cresol (mainly generated from tyrosine), and trimethylamine (mainly generated from choline, L-carnitine, and betaine), which further enters the liver via the hepatic portal vein to generate IS (metabolised by CYP2E1 and SULT1A), pCS (metabolised by SULT1A1), and TMAO (metabolised by FMO3) under the metabolic effect of the metabolic enzymes in liver (Lim et al., 2021). Therefore, this study focused on gut-derived uremic toxins to investigate the mechanism of TGT to improve CKD. Non-target metabolomics was used to explore the changes in plasma metabolites and found that amino acids, indole and their derivatives were significantly upregulated after the CKD rat model was established. Metabolic pathway analysis was performed for the differential metabolites, which showed that tryptophan metabolism and phenylalanine metabolism were significantly upregulated in the model group compared to the control group. After TGT intervention, a large number of metabolites were significantly downregulated, especially the plasma levels of amino acids, indole and their derivatives were completely downregulated. Thus, we inferred that the mechanism by which TGT ameliorates CKD contains the regulation of gut-derived uremic toxins *in vivo*. From our data, it was shown that TGT significantly reduced plasma levels of IS and pCS but increased TMAO levels by 28.1% with no significant. Meanwhile, AEF significantly increased TMAO plasma levels and did not show the significant reduction ability on plasma levels of IS and pCS. CKD patients with greater serum TMAO levels were identified to have a higher risk of all-cause mortality in a study of adult individuals undergoing coronary angiography (Lim et al., 2021). Additionally, *in vivo* research has demonstrated that a high-fat diet or dietary supplements of choline or TMAO increase the expression of pro-fibrotic genes and kidney damage markers, as well as promote tubulointerstitial fibrosis (Sun et al., 2017; Lim et al., 2021). As a result, AEF may worsen the pathophysiology of CKD by causing the body to accumulate TMAO.

The 16S rRNA study of the intestinal bacteria in the rat faeces showed that TGT strongly influenced the intestinal flora due to its distinct microbial spectrum. The TGT group’s gut flora clustering in the PCA was far from the control group and overlapped with the model and AEF groups, indicating the intricate modulation of gut microbiota by TGT and maybe the mechanism of CKD treatment. Among the uremic toxins-producer in gut microbiota, indole-producing strains mainly belong to the *f_*Bacteroidaceae, *f_*Enterobacteriaceae, and *f_*Pasteurellaceae, which codes for TIL to mediate the conversion of tryptophan to indole (Harris and Phillips, 2013; Devlin et al., 2016). However, *p*-cresol-producing strains mainly belong to *f_*Enterococcaceae, *f_*Clostridiaceae*,* and *f_*Staphylococcaceae, which codes for tyrosine amino-transferase to mediate the degradation of tyrosine (Kikuchi et al., 2019; Zhu et al., 2020). Notably, TMA-producing strains are widely distributed in various phyla, such as *f_*Clostridiaceae and *f_*Enterobacteriaceae (Ji et al., 2021), which code for TMA-lyase enzyme complexes CutC/D (Craciun and Balskus, 2012), CntA/B (Koeth et al., 2014), and YeaW/X (Zhu et al., 2014) that mediate the conversion of choline, L-carnitine, and betaine to TMA, respectively. In this study, TGT has shown a significant decrease in IS and pCS plasma levels. The contents of toxin precursors in plasma and faeces were measured, and it was found that tryptophan was restored to normal levels in the TGT group. In addition, the non-target metabolome showed a significant reduction in plasma levels of indole and its derivatives after TGT intervention. Based on our data, it was found that TGT reduced the level of IS in plasma by regulating the tryptophan-indole-IS pathway. TGT significantly regulated the abundance of *g_Bacteroides* and *g_Rodentibacter* in CKD rats, which belong to the *f_*Bacteroidaceae and *f_*Pasteurellaceae, respectively. Thus, we inferred that TGT may have reduced the coding of TIL, which significantly inhibited the production of indole. In our previous study, by the human liver microsomes with cocktail methods, we found that celastrol can uncompetitively inhibit CYP2E1 activity (Jin et al., 2015). This may have inhibited the conversion of indole to IS, which reduced the production of IS.

Furthermore, the kidney contributes significantly to the removal of various endogenous poisons and medications through the unique processes of active tubular secretion and passive glomerular filtration (Lepist and Ray, 2016). And renal transport pathways mainly involve apical and basolateral transporters acting in concert (George et al., 2017; Müller et al., 2018). It was reported that IS and pCS, as protein-bound toxins, are often uptaken via organic anion transporters (OAT) one and 3, then effluxed into the urine by OAT4 (Enomoto et al., 2002; Miyamoto et al., 2011). And TMAO, as a free water-soluble low-molecular-weight toxin, is uptaken by organic cation transporter (OCT) two and then effluxed to the urine by multidrug and toxin extrusion proteins (MATE) 1 (Gessner et al., 2018). Currently, *tripterygium* glycosides have been shown to damage the OAT system that is localised at the proximal tubule, especially the S2 segment, resulting in a substantial decrease in the mRNA expression of OAT1 and OAT3 (Dan et al., 2008), which affects IS and pCS uptake. We speculated that AEF may impair the apical and basolateral transporters, OCT2 and MATE1, thereby reducing urinary excretion of TMAO. And the content of TMAO in the plasma was also significantly increased by AEF. Furthermore, there was a significantly increased urinary excretion of pCS after TGT treatment *in vivo*. Additionally, TGT downregulated the amount of pCS in the plasma. Therefore, we can conclude that TGT may have an impact on pCS excretion. To fully understand how TGT controls the excretion of uremic toxins, more research was required. In summary, elucidating the efficacy and toxicity fractions and their mechanisms in the treatment of CKD with TGT is crucial for enhancing efficacy, controlling toxicity, and advancing its precision medicine in clinical practice.

## 5 Conclusion

**FIGURE 7:**
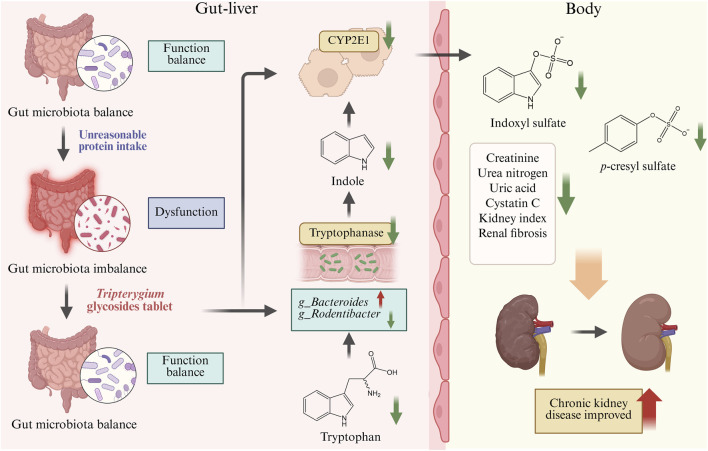
TGT can improve CKD via the gut-liver-kidney axis by regulating the metabolism of gut-derived uremic toxins such as the tryptophan-indole-indoxyl sulfate pathway.

**FIGURE 8:**
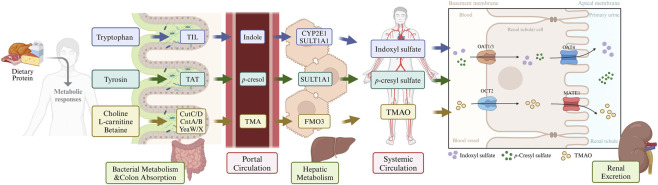
The gut-liver-kidney axis of absorption, metabolism and excretion pathways of gut-derived uremic toxins.

In this study, TGT was split into AEF and NAEF based on the constituents knock-out/knock-in technology. After that, studies on TGT’s therapeutic effect on CKD showed that it greatly improved renal filtration function, histomorphology, and renal fibrosis in CKD rats while also lowering plasma levels of creatinine, urea nitrogen, and uric acid. Non-target metabolomics and 16S rRNA showed that TGT improved the structure of the intestinal flora, particularly regulating the abundance of *g_Bacteroides* and *g_Rodentibacter*, and reduced the levels of indole and amino acid derivatives *in vivo*. Moreover, as shown in Figure 7, we found that TGT decreased gut-derived uremic toxins, IS and pCS, and the mechanism may be related to the inhibition of the tryptophan-indole-IS pathway and the improvement of the urinary excretion of pCS. Notably, AEF increased the plasma levels of creatinine, urea nitrogen, cystatin C, and TMAO and aggravated the impairment of renal function. This study clarified that AEF is the principal toxic fraction and NAEF is the beneficial fraction of TGT. Moreover, it is the first to demonstrate that TGT ameliorates CKD through the gut–liver–kidney axis (Figure 8), providing new insights on CKD treatment.

## Data Availability

The original contributions presented in the study are included in the article/Supplementary Material, further inquiries can be directed to the corresponding authors.
