# Supplementary material for: Tripterygium glycosides tablet ameliorates chronic kidney disease via gut-derived uremic toxins reduction in rats: alkaloids as major toxic ingredients
Source: Front Pharmacol. 2026 Jul 3;17:1739319. doi: 10.3389/fphar.2026.1739319 (PMC13375623; doi:10.3389/fphar.2026.1739319)
Supplement: Supplementary file 1 [file Supplementaryfile1.docx]

Supplementary Material

**Supplementary material for**

Original article

*Tripterygium* glycosides tablet ameliorates chronic kidney disease via gut-derived uremic toxins reduction in rats: alkaloids as major toxic ingredients.

He Gong, Sixian Chen, Zhuohua Huang, Hong Yuan, Jiang Ma^*^, Xin He^*^.

**Supplementary material includes:**

1. Supporting tables S1–S10 (Table S1–S10)

2. Supporting figures S1–S9 (Fig. S1–S9)

1. Supporting tables

**Table S1** MRM method parameter of the LC-MS/MS method for uremic toxins and precursors.

| Method | Compounds | （+/-） | Q1 Mass (Da) | Q3 Mass (Da) | Rt (min) | Q1 Pre (V) | CE (V) | Q3 Pre (V) |
| --- | --- | --- | --- | --- | --- | --- | --- | --- |
| C_18_  method | Indoxyl sulfate | - | 212.10 | 79.95 | 0.586 | 15 | 21 | 20 |
|  | *p*-cresyl sulfate | - | 187.10 | 107.00 | 0.659 | 15 | 18 | 18 |
|  | HCT (IS) | - | 296.00 | 269.00 | 0.939 | 13 | 17 | 13 |
| Hilic method | Choline | + | 104.40 | 60.15 | 1.548 | -19 | -21 | -23 |
|  | L-carnitine | + | 162.15 | 60.05 | 2.034 | -19 | -17 | -23 |
|  | Betaine | + | 118.10 | 58.15 | 2.096 | -13 | -27 | -22 |
|  | Tryptophan | + | 205.25 | 188.05 | 1.432 | -14 | -10 | -19 |
|  | Tyrosine | + | 182.35 | 136.20 | 1.485 | -13 | -12 | -29 |
|  | TMAO | + | 76.20 | 58.10 | 1.736 | -15 | -22 | -23 |
|  | FVal (IS) | + | 136.30 | 70.05 | 1.483 | -10 | -15 | -27 |

IS: internal standard; HCT: hydrochlorothiazide; TMAO: trimethylamine *N*-oxide; FVal: 3-Fluoro-DL-valine; Rt: retention time.

**Table S2** Results of repeatability of HPLC fingerprinting (*n* = 6).

| Peak No. | Rt (Mean) | Rt (RSD%) | Area (Mean) | Area (RSD%) |
| --- | --- | --- | --- | --- |
| 1 | 75.30 | 0.26 | 161337.00 | 3.75 |
| 2 | 94.77 | 0.14 | 838020.83 | 4.42 |
| 3 | 107.36 | 0.12 | 252045.67 | 3.67 |
| 4 | 116.55 | 0.11 | 36415.33 | 4.25 |
| 5 | 132.62 | 0.13 | 5061311.17 | 2.08 |
| 6 | 183.51 | 0.09 | 9754415.00 | 1.16 |

Rt: retention time (min); RSD: relative standard deviation.

**Table S3** Results of precision of HPLC fingerprinting (*n* = 6).

| Peak No. | Rt (Mean) | Rt (RSD%) | Area (Mean) | Area (RSD%) |
| --- | --- | --- | --- | --- |
| 1 | 75.37 | 0.18 | 151603.00 | 3.27 |
| 2 | 94.76 | 0.11 | 833135.50 | 4.48 |
| 3 | 107.33 | 0.09 | 243051.67 | 3.54 |
| 4 | 116.57 | 0.10 | 37096.50 | 3.78 |
| 5 | 132.68 | 0.09 | 5196132.60 | 2.49 |
| 6 | 183.53 | 0.06 | 10008142.67 | 0.55 |

Rt: retention time (min); RSD: relative standard deviation.

**Table S4** Results of stability of HPLC fingerprinting (24 h at 25 ℃, *n* = 6).

| Peak No. | Rt (Mean) | Rt (RSD%) | Area (Mean) | Area (RSD%) |
| --- | --- | --- | --- | --- |
| 1 | 75.35 | 0.17 | 159537.00 | 1.02 |
| 2 | 94.75 | 0.11 | 845501.14 | 3.67 |
| 3 | 107.32 | 0.09 | 261080.29 | 4.77 |
| 4 | 116.55 | 0.10 | 37710.00 | 4.64 |
| 5 | 132.66 | 0.10 | 5250249.14 | 2.78 |
| 6 | 183.50 | 0.07 | 9995398.71 | 0.61 |

Rt: retention time (min); RSD: relative standard deviation.

**Table S5** Method sensitivity and linear range.

| C_18_ - method | | LLOD (ng/mL) | LLOQ (ng/mL) | Linear range (ng/mL) | Calibration Curve | | *r*^2^ |
| --- | --- | --- | --- | --- | --- | --- | --- |
| Plasma | Indoxyl sulfate | 5 | 10 | 10 - 10000 | Y = 0.310404 X + 0.00285566 | | 0.999 |
|  | *p*-cresyl sulfate | 1 | 2 | 2 - 10000 | Y = 2.86141 X + 0.652166 | | 0.998 |
| Urine | Indoxyl sulfate | 2 | 5 | 5 - 10000 | Y = 0.261620 X - 0.0875919 | | 0.996 |
|  | *p*-cresyl sulfate | 1 | 2 | 2 - 10000 | | Y = 5.85912 X - 0.0663267 | 0.999 |

| Hilic - method | | LLOD (ng/mL) | LLOQ (ng/mL) | Linear range (ng/mL) | Calibration Curve | *r*^2^ |
| --- | --- | --- | --- | --- | --- | --- |
| Plasma | Choline | 2 | 5 | 5 – 10000 | Y = 30.5251 X + 19.8805 | 0.997 |
|  | L-carnitine | 2 | 5 | 5 – 10000 | Y = 7.89512 X + 0.307661 | 0.997 |
|  | Betaine | 1 | 2 | 2 – 10000 | Y = 15.8716 X + 10.3173 | 0.989 |
|  | Tryptophan | 2 | 5 | 5 – 10000 | Y = 7.92363 X - 0.916365 | 0.999 |
|  | Tyrosine | 5 | 10 | 10 – 10000 | Y = 0.282068 X + 0.0494042 | 0.999 |
|  | TMAO | 1 | 2 | 2 – 10000 | Y = 32.1664 X + 0.0896025 | 0.998 |
| Urine | TMAO | 1 | 2 | 2 – 10000 | Y = 5.30573 X + 2.60216 | 0.994 |
| Faeces | Choline | 1 | 2 | 2 – 10000 | Y = 65.3746 X + 4.80279 | 0.997 |
|  | L-carnitine | 1 | 2 | 2 – 10000 | Y = 4.43691 X + 0.360236 | 0.997 |
|  | Betaine | 1 | 2 | 2 – 10000 | Y = 55.3368 X + 0.0299284 | 0.996 |
|  | Tryptophan | 1 | 2 | 2 – 10000 | Y = 3.81156 X - 0.140193 | 0.996 |
|  | Tyrosine | 20 | 40 | 40 - 10000 | Y = 0.618279 X - 0.0519505) | 0.996 |

LLOD: lower limit of determination; LLOQ: lower limit of quantification.

**Table S6** Accuracy and recision for plasma (*n* = 6).

|  | | Intra-day (*n* = 6) | | | | | | Inter-day (*n* = 3) | | | | | |
| --- | --- | --- | --- | --- | --- | --- | --- | --- | --- | --- | --- | --- | --- |
|  |  | Accuracy (RE%) | | | RSD (%) | | | Accuracy (RE%) | | | RSD (%) | | |
|  |  | LQC | MQC | HQC | LQC | MQC | HQC | LQC | MQC | HQC | LQC | MQC | HQC |
| Hilic method | Choline | -1.90 | -5.19 | 10.31 | 2.03 | 10.68 | 6.87 | 0.22 | 3.47 | 2.75 | 11.96 | 11.76 | 11.28 |
|  | L-carnitine | -6.06 | 4.29 | 2.72 | 4.36 | 5.14 | 1.16 | -6.04 | 1.38 | -9.70 | 9.50 | 9.81 | 4.99 |
|  | Betaine | 8.70 | -4.11 | -4.13 | 3.54 | 3.44 | 1.08 | 9.08 | 12.24 | 0.15 | 4.99 | 10.94 | 10.09 |
|  | Tryptophan | -9.89 | 12.95 | 3.51 | 3.05 | 3.16 | 8.14 | -2.55 | -14.49 | 5.22 | 10.52 | 11.23 | 14.23 |
|  | Tyrosine | 11.89 | -1.44 | -5.30 | 8.63 | 6.26 | 10.22 | -4.03 | -3.75 | -11.30 | 12.14 | 13.79 | 14.19 |
|  | TMAO | 5.85 | -8.22 | -6.66 | 5.99 | 1.80 | 4.32 | 9.72 | -4.68 | -2.50 | 4.63 | 3.78 | 4.55 |
| C_18_  method | Indoxyl sulfate | -10.50 | 6.84 | 2.02 | 12.60 | 10.18 | 11.94 | -3.76 | 12.94 | -5.95 | 9.85 | 9.08 | 6.20 |
|  | *p*-cresyl sulfate | -1.96 | 7.59 | -5.02 | 6.58 | 7.33 | 6.66 | 12.86 | 13.56 | 5.74 | 4.34 | 5.27 | 1.95 |

RSD: relative standard deviation; LQC: low quality control; MQC: middle quality control; HQC: high quality control.

**Table S7** Accuracy and recision for urine (*n* = 6).

|  | | Intra-day (*n* = 6) | | | | | | Inter-day (*n* = 3) | | | | | |
| --- | --- | --- | --- | --- | --- | --- | --- | --- | --- | --- | --- | --- | --- |
|  |  | Accuracy (RE%) | | | RSD (%) | | | Accuracy (RE%) | | | RSD (%) | | |
|  |  | LQC | MQC | HQC | LQC | MQC | HQC | LQC | MQC | HQC | LQC | MQC | HQC |
| Hilic  method | TMAO | -0.25 | 3.84 | 3.15 | 4.14 | 3.77 | 5.84 | 0.83 | 4.51 | -14.23 | 4.33 | 2.75 | 5.66 |
| C_18_  method | Indoxyl sulfate | -8.47 | 3.55 | 6.73 | 2.98 | 4.13 | 0.80 | 1.75 | 1.22 | -6.65 | 3.67 | 2.97 | 2.08 |
|  | *p*-cresyl sulfate | 3.69 | 3.87 | -2.95 | 2.56 | 2.99 | 1.74 | 10.59 | 2.54 | -5.58 | 3.14 | 2.24 | 1.95 |

RSD: relative standard deviation; LQC: low quality control; MQC: middle quality control; HQC: high quality control.

**Table S8** Accuracy and recision for faeces (*n* = 6).

|  | | Intra-day (*n* = 6) | | | | | | Inter-day (*n* = 3) | | | | | |
| --- | --- | --- | --- | --- | --- | --- | --- | --- | --- | --- | --- | --- | --- |
|  |  | Accuracy (RE%) | | | RSD (%) | | | Accuracy (RE%) | | | RSD (%) | | |
|  |  | LQC | MQC | HQC | LQC | MQC | HQC | LQC | MQC | HQC | LQC | MQC | HQC |
| Hilic method | Choline | -5.73 | 4.31 | 9.17 | 7.66 | 8.50 | 5.67 | 6.92 | -8.33 | -4.92 | 12.08 | 12.12 | 13.41 |
|  | L-carnitine | 10.63 | 6.12 | 7.92 | 3.75 | 13.41 | 9.77 | -4.31 | 10.55 | 11.94 | 13.47 | 11.78 | 12.45 |
|  | Betaine | 2.21 | -9.27 | -8.89 | 4.37 | 10.40 | 3.94 | 3.91 | -9.99 | 8.28 | 5.29 | 5.73 | 6.69 |
|  | Tryptophan | -10.69 | 13.16 | -1.93 | 2.52 | 4.66 | 3.59 | 0.36 | 2.40 | -4.79 | 10.07 | 8.25 | 5.67 |
|  | Tyrosine | 6.71 | -0.38 | 3.90 | 7.40 | 10.27 | 8.02 | -9.64 | -5.53 | 7.60 | 12.04 | 12.63 | 13.59 |

RSD: relative standard deviation; LQC: low quality control; MQC: middle quality control; HQC: high quality control.

**Table S9** Extraction recovery (*n* = 6).

| C_18_ - method | | Accuracy (RE%) | | | RSD (%) | | |
| --- | --- | --- | --- | --- | --- | --- | --- |
|  |  | LQC | MQC | HQC | LQC | MQC | HQC |
| Plasma | Indoxyl sulfate | 10.16 | 9.99 | 4.66 | 10.16 | 9.99 | 4.66 |
|  | *p*-cresyl sulfate | 3.77 | 3.96 | 1.92 | 3.77 | 3.96 | 1.92 |
| Urine | Indoxyl sulfate | -3.39 | 11.28 | 1.07 | 2.31 | 0.97 | 2.21 |
|  | *p*-cresyl sulfate | 6.55 | -1.78 | -6.80 | 2.14 | 1.96 | 1.07 |

| Hilic - method | | Accuracy (RE%) | | | RSD (%) | | |
| --- | --- | --- | --- | --- | --- | --- | --- |
|  |  | LQC | MQC | HQC | LQC | MQC | HQC |
| Plasma | Choline | 10.95 | -2.57 | 13.01 | 3.42 | 10.52 | 6.98 |
|  | L-carnitine | -7.27 | 5.25 | 6.45 | 4.53 | 2.76 | 7.15 |
|  | Betaine | -4.85 | -9.08 | 3.97 | 4.18 | 5.29 | 3.77 |
|  | Tryptophan | 10.95 | -13.30 | 0.19 | 4.02 | 3.01 | 5.35 |
|  | Tyrosine | -4.18 | 6.83 | -7.92 | 7.33 | 8.99 | 5.64 |
|  | TMAO | 12.40 | -4.02 | 9.43 | 4.34 | 5.02 | 0.94 |
| Urine | TMAO | -4.90 | 10.27 | -7.92 | 4.62 | 1.24 | 2.04 |
| Faeces | Choline | 8.89 | -5.41 | -11.92 | 4.25 | 9.56 | 2.24 |
|  | L-carnitine | -11.94 | 10.23 | 5.06 | 3.37 | 12.65 | 1.15 |
|  | Betaine | -12.06 | 7.92 | -4.50 | 4.69 | 9.21 | 4.23 |
|  | Tryptophan | 3.68 | -9.64 | -0.66 | 1.93 | 2.92 | 1.25 |
|  | Tyrosine | 8.12 | -5.60 | 6.82 | 3.11 | 8.31 | 2.86 |

RSD: relative standard deviation; LQC: low quality control; MQC: middle quality control; HQC: high quality control.

**Table S10** Sample stability (*n* = 6).

|  |  | Processed sample stability  (after 24 h at 4 ℃) | | | | Bench-top stability  (after 12 at room temperature) | | | | Two freeze–thaw cycle stability | | | |
| --- | --- | --- | --- | --- | --- | --- | --- | --- | --- | --- | --- | --- | --- |
| C_18_ - method | | Accuracy (RE%) | | RSD (%) | | Accuracy (RE%) | | RSD (%) | | Accuracy (RE%) | | RSD (%) | |
|  |  | LQC | HQC | LQC | HQC | LQC | HQC | LQC | HQC | LQC | HQC | LQC | HQC |
| Plasma | Indoxyl sulfate | -2.77 | 5.80 | 9.48 | 6.63 | -2.86 | 5.28 | 11.11 | 5.37 | 4.11 | -8.74 | 10.31 | 5.66 |
|  | *p*-cresyl sulfate | -4.94 | 6.65 | 3.66 | 1.96 | 12.04 | -1.38 | 3.33 | 1.79 | 12.72 | -0.93 | 3.52 | 2.08 |
| Urine | Indoxyl sulfate | 4.51 | 1.66 | 2.38 | 3.97 | 11.50 | 1.15 | 3.13 | 2.21 | -4.26 | 1.46 | 3.26 | 1.98 |
|  | *p*-cresyl sulfate | -3.11 | 8.17 | 1.51 | 2.14 | -3.73 | 5.88 | 1.99 | 1.81 | 13.54 | 8.76 | 2.06 | 1.81 |

|  |  | Processed sample stability  (after 24 h at 4 ℃) | | | | Bench-top stability  (after 12 at room temperature) | | | | Two freeze–thaw cycle stability | | | |
| --- | --- | --- | --- | --- | --- | --- | --- | --- | --- | --- | --- | --- | --- |
| Hilic - method | | Accuracy (RE%) | | RSD (%) | | Accuracy (RE%) | | RSD (%) | | Accuracy (RE%) | | RSD (%) | |
|  |  | LQC | HQC | LQC | HQC | LQC | HQC | LQC | HQC | LQC | HQC | LQC | HQC |
| Plasma | Choline | 11.79 | 2.95 | 3.08 | 3.95 | 7.58 | -4.62 | 4.45 | 4.99 | 6.10 | 5.68 | 5.22 | 9.15 |
|  | L-carnitine | -7.68 | -0.58 | 3.01 | 1.79 | 2.51 | -7.99 | 7.06 | 10.86 | 2.63 | -12.86 | 8.03 | 5.46 |
|  | Betaine | 3.12 | -2.38 | 5.50 | 2.19 | 10.24 | 4.83 | 4.45 | 11.61 | -7.87 | 11.19 | 7.10 | 12.74 |
|  | Tryptophan | 10.47 | 4.03 | 10.56 | 8.33 | -8.60 | 12.40 | 10.04 | 10.80 | 10.67 | -3.68 | 10.64 | 11.20 |
|  | Tyrosine | -7.43 | -2.97 | 13.61 | 8.05 | 11.99 | -4.89 | 2.07 | 12.69 | 8.82 | 9.03 | 9.90 | 14.46 |
|  | TMAO | -9.17 | 10.10 | 5.61 | 2.33 | -7.11 | -8.79 | 4.35 | 1.34 | -11.58 | -6.45 | 6.21 | 5.05 |
| Urine | TMAO | -0.83 | -6.90 | 1.43 | 1.77 | 4.57 | -1.46 | 3.86 | 5.62 | 1.76 | 10.17 | 3.75 | 7.17 |
| Faeces | Choline | -7.06 | 11.12 | 7.89 | 3.85 | 4.61 | 12.89 | 13.50 | 10.65 | 5.14 | -3.85 | 11.94 | 11.08 |
|  | L-carnitine | -3.68 | -13.07 | 9.68 | 2.52 | 6.09 | -7.35 | 8.37 | 12.15 | -11.97 | 9.33 | 10.77 | 11.80 |
|  | Betaine | 14.15 | 5.83 | 12.11 | 6.42 | 3.41 | -10.26 | 14.00 | 7.73 | -7.75 | 4.99 | 8.98 | 7.12 |
|  | Tryptophan | -8.38 | 2.74 | 5.02 | 1.69 | -5.79 | 13.29 | 8.76 | 5.08 | 14.45 | -2.07 | 7.74 | 4.45 |
|  | Tyrosine | 6.71 | 9.27 | 9.05 | 8.06 | 11.37 | 2.65 | 12.76 | 11.60 | 6.83 | -0.56 | 12.22 | 13.86 |

RSD: relative standard deviation; LQC: low quality control; MQC: middle quality control; HQC: high quality control.

2. Supporting figures


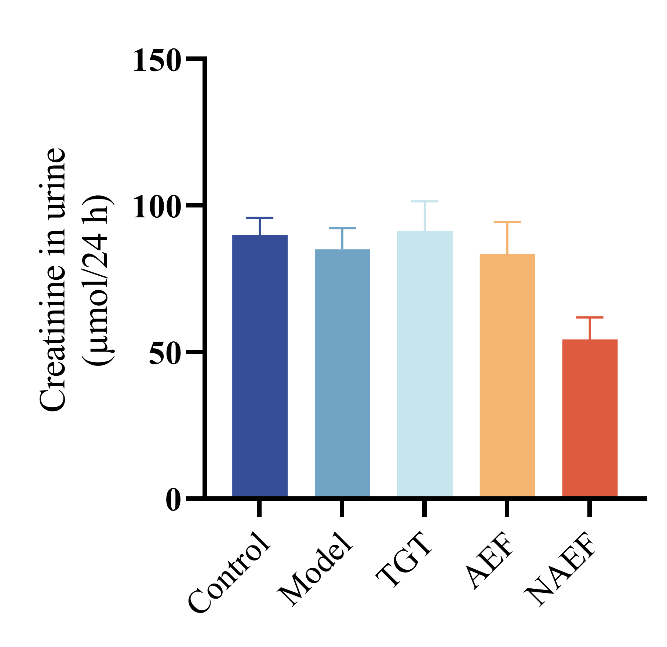


**Fig. S1.** The 24 h cumulative excretion of creatinine in urine. The data are shown as mean ± SD, *n* = 6.


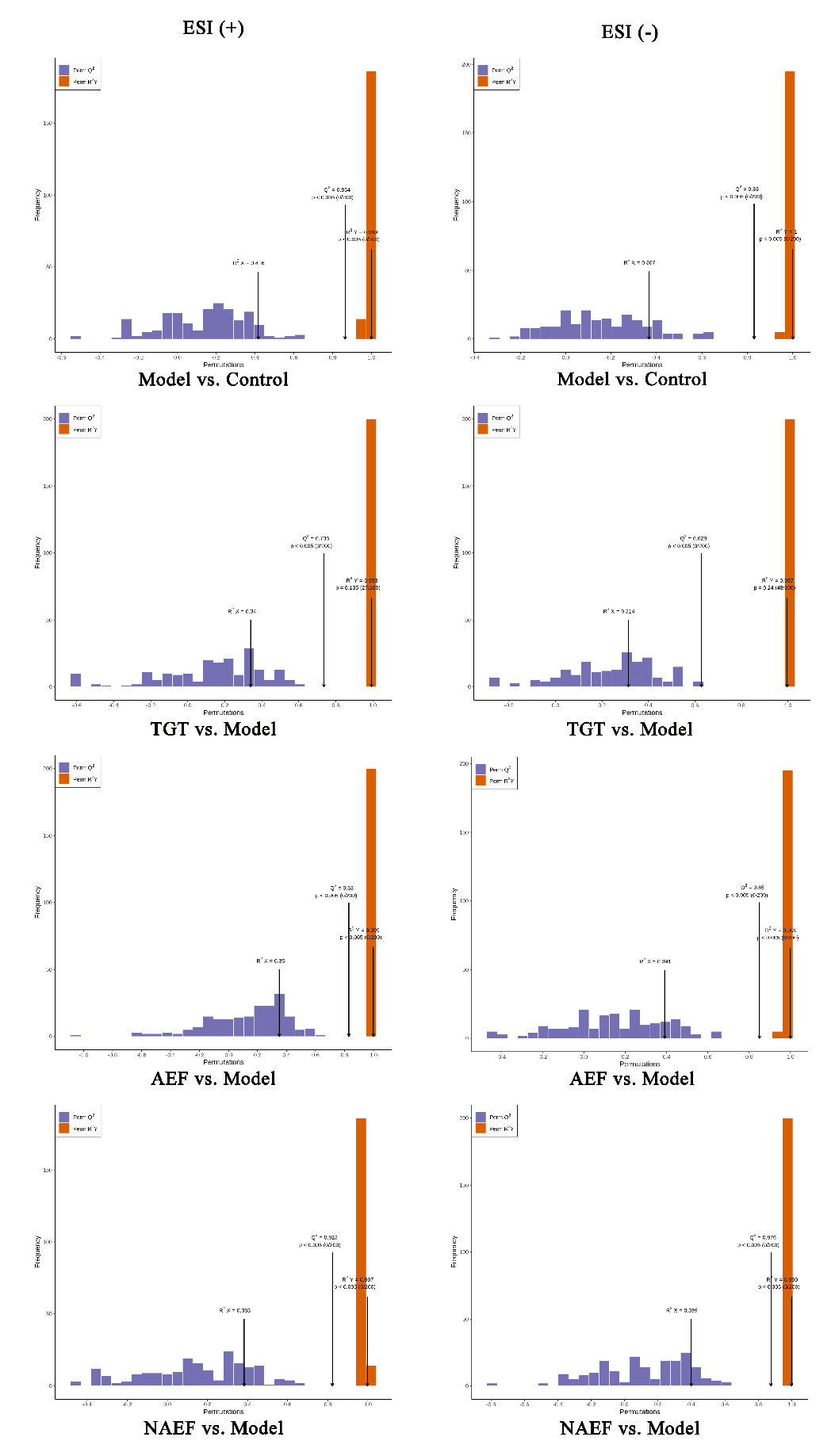


**Fig. S2.** OPLS-DA models and permutation tests in the plasma metabolome. R^2^ Y and Q^2^ values > 0.5 mean that the models have good interpretability and predictability, *P* value< 0.05 means that the models are not overfitted.


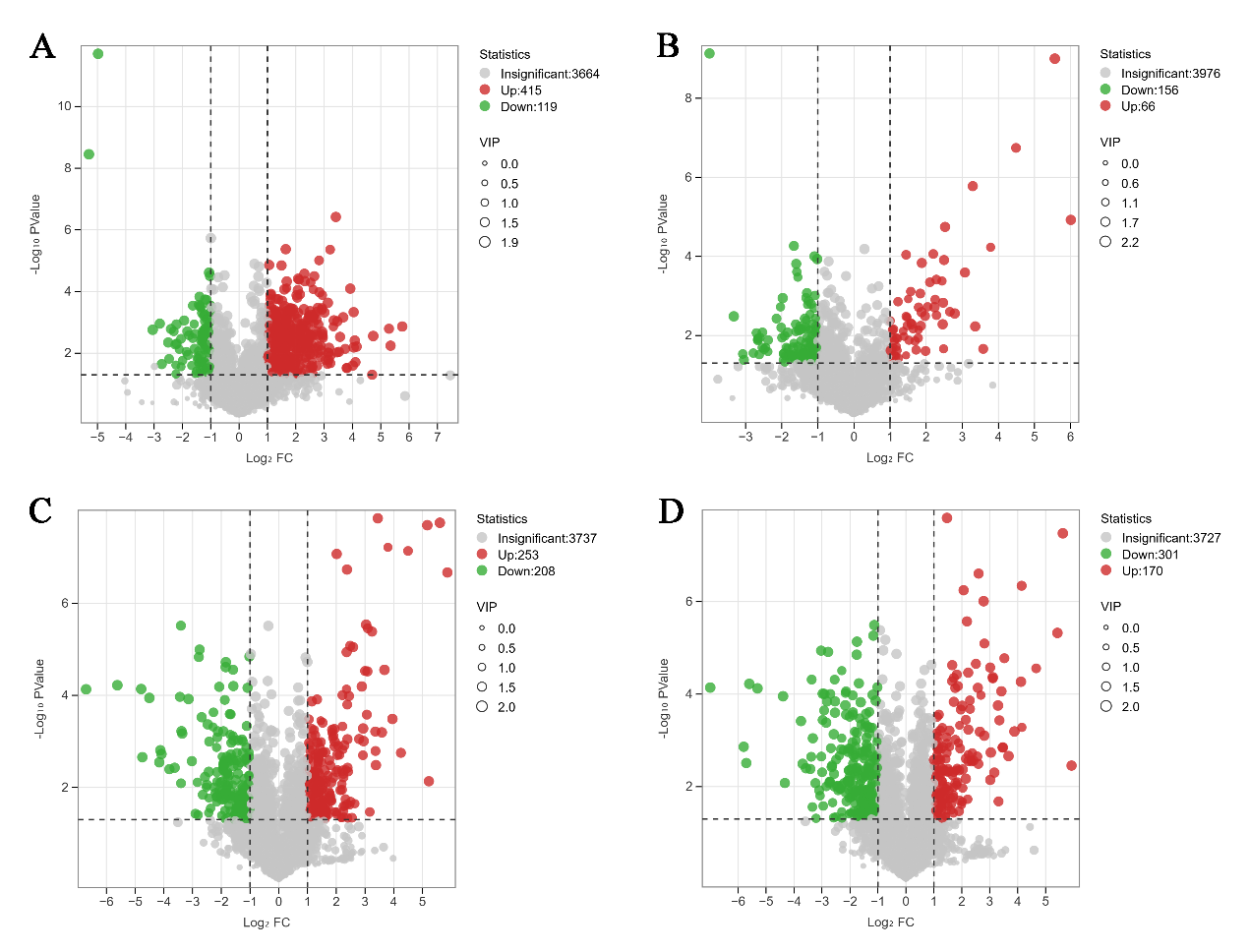


**Fig. S3.** Volcano plot analysis of the differential metabolites in plasma. (A) The Model group vs. the Control group. (B) The TGT group vs. the Model group. (C) The AEF group vs. the Model group. (D) The NAEF group vs. the Model group.

**
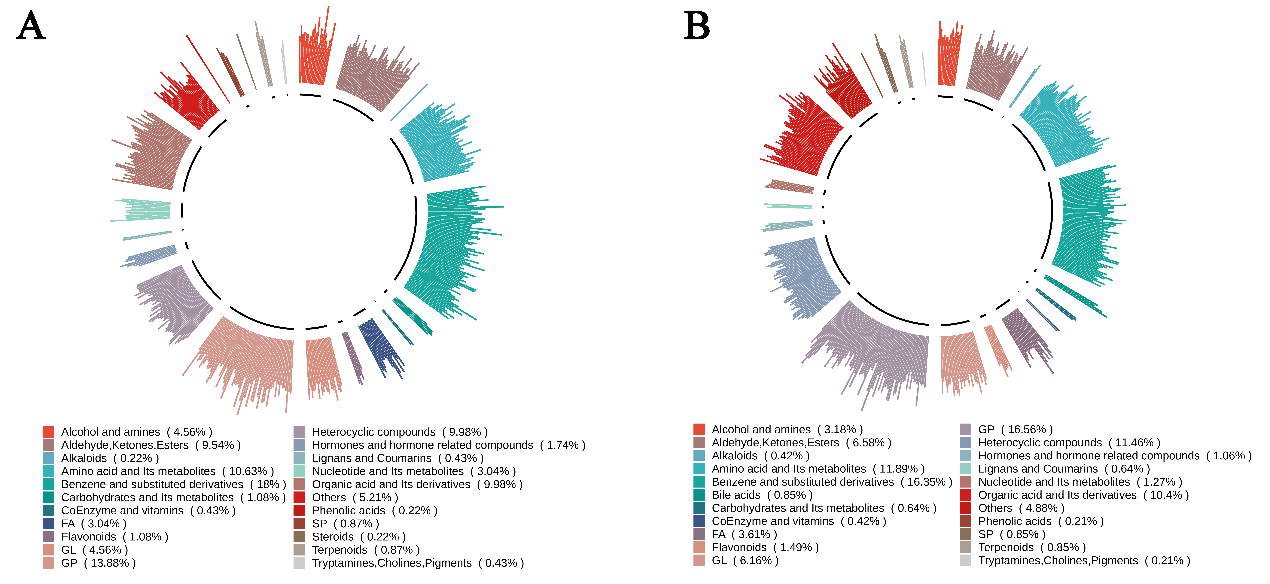
**

**Fig. S4.** The primary classification of differential metabolites. (A) The AEF group vs. the Model group. (B) The NAEF group vs. the Model group.

**
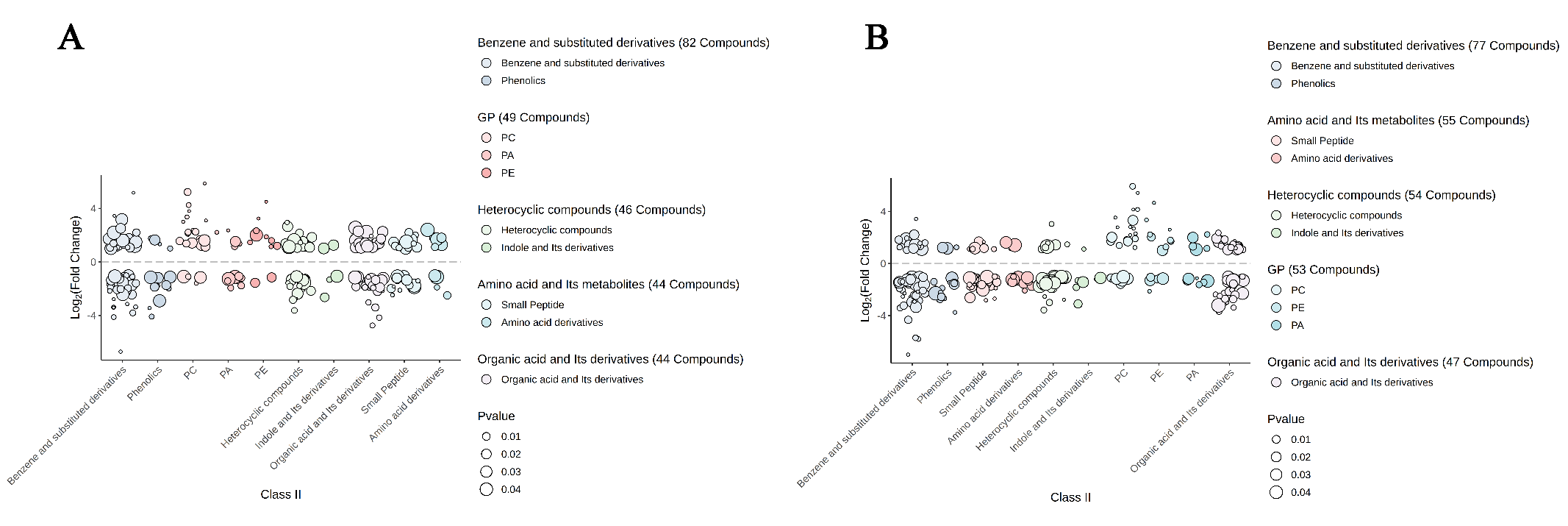
**

**Fig. S5.** The top 5 differential metabolites. (A) The AEF group vs. the Model group. (B) The NAEF group vs. the Model group.


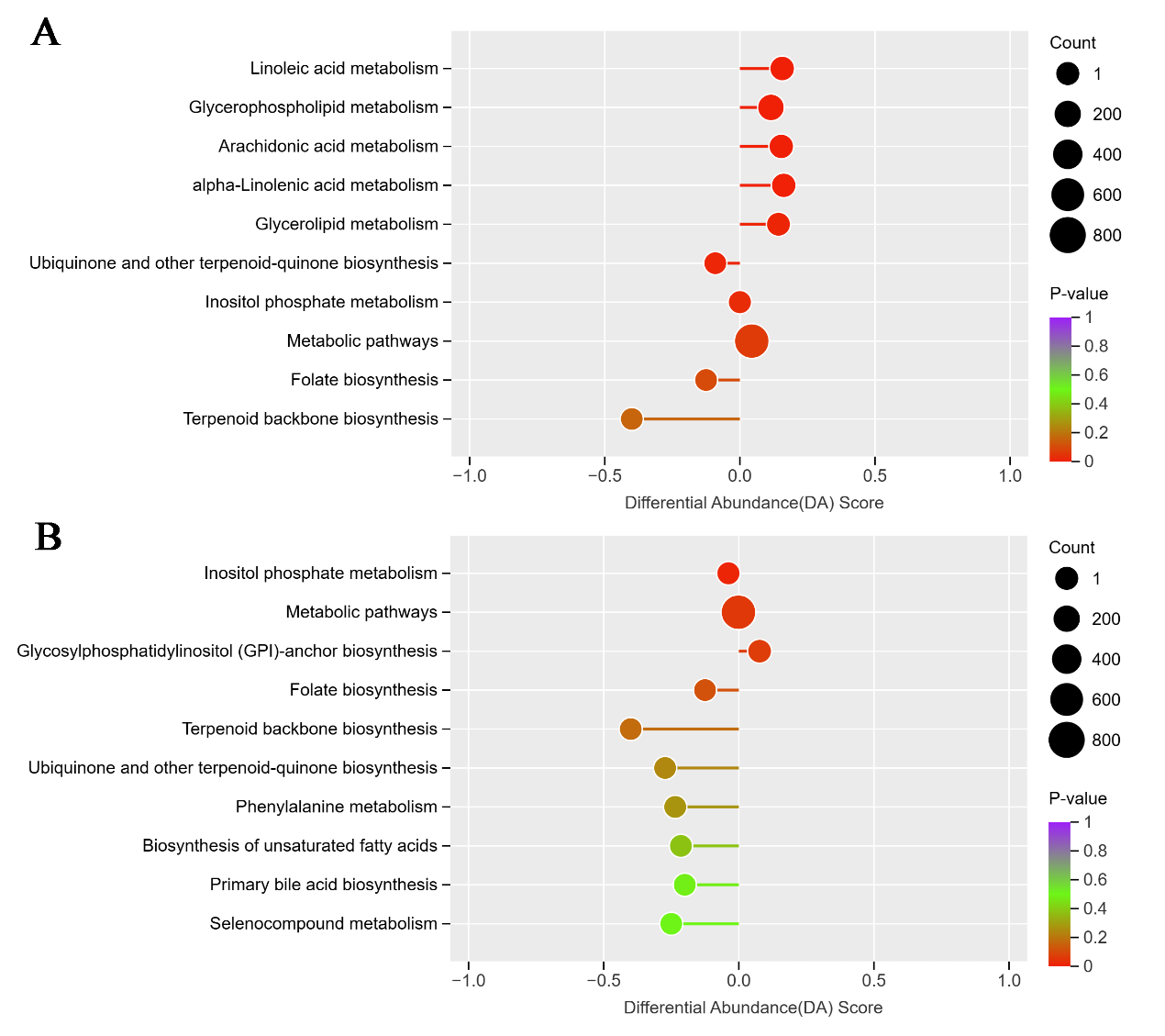


**Fig. S6.** Pathway analysis of differential metabolites. (A) The AEF group vs. the Model group. (B) The NAEF group vs. the Model group.


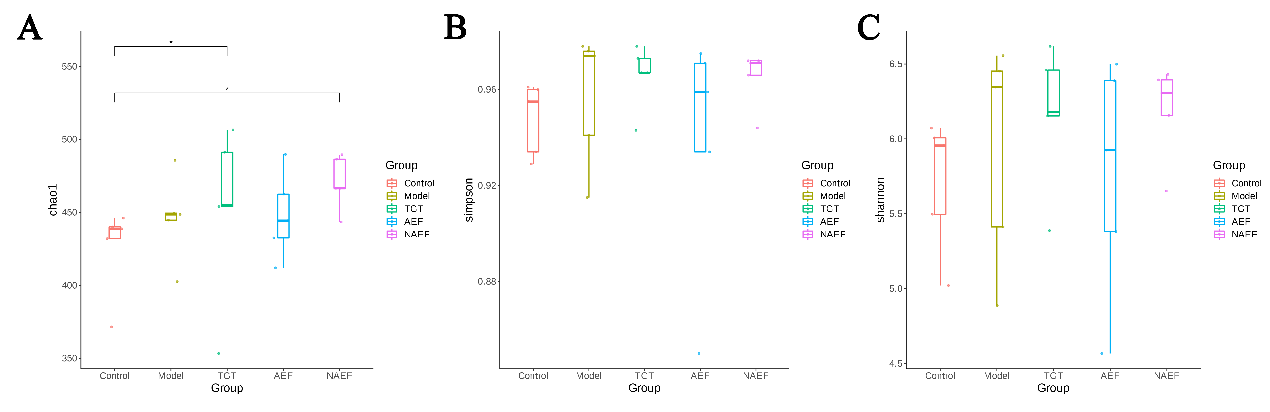


**Fig. S7.** The *α*-diversity of gut bacteria in CKD model. (A) Chao 1 index. (B) Simpson index. (C) Shannon index. The data are shown as mean ± SD, ^*^*p* < 0.05, *n* = 5.

**
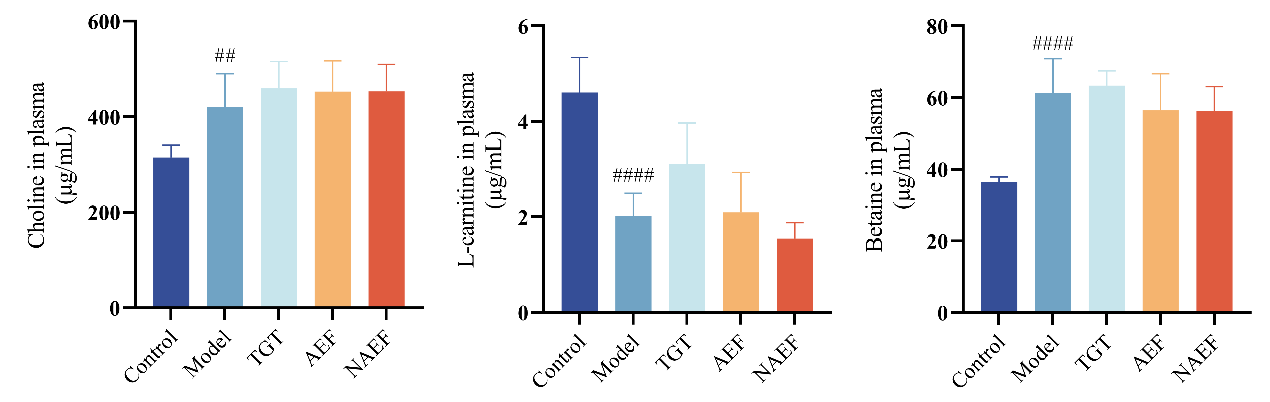
**

**Fig. S8.** The content of choline, L-carnitine and betaine in plasma. The data are shown as mean ± SD, compared with control group, ^##^*p* < 0.01, ^####^*p* < 0.000, *n* = 6.

**
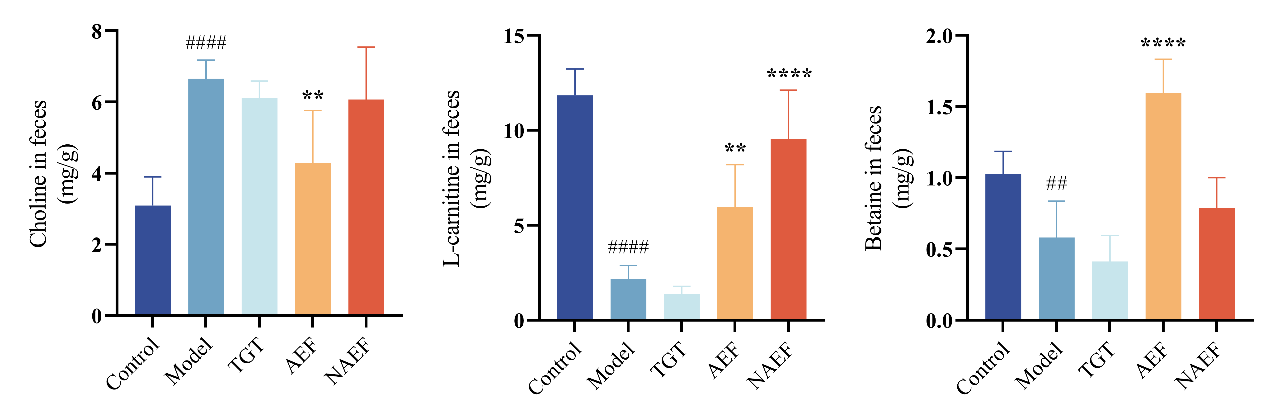
**

**Fig. S9.** The content of choline, L-carnitine and betaine in faeces. The data are shown as mean ± SD, compared with control group, ^##^*p* < 0.01, ^####^*p* < 0.0001; compared with model group, ^**^*p* < 0.01, ^****^*p* < 0.0001, *n* = 6.
